# Remote-controllable bone-targeted delivery of estradiol for the treatment of ovariectomy-induced osteoporosis in rats

**DOI:** 10.1186/s12951-021-00976-4

**Published:** 2021-08-18

**Authors:** Yuanyuan Guo, Yongwei Liu, Chen Shi, Tingting Wu, Yongzhi Cui, Siyuan Wang, Ping Liu, Xiaobo Feng, Yu He, Dehao Fu

**Affiliations:** 1 Department of Pharmacy, Liyuan Hospital, Tongji Medical College, Huazhong University of Science and Technology, Wuhan, China; 2 Department of Orthopedics, Union Hospital, Tongji Medical College, Huazhong University of Science and Technology, Wuhan, China; 3 Department of Pharmacy, Union Hospital, Tongji Medical College, Huazhong University of Science and Technology, Wuhan, China; 4 Department of Orthopedics, Shanghai General Hospital, Shanghai Jiaotong University, School of Medicine, Shanghai, China; 5 Department of Orthopedics, Liyuan Hospital, Tongji Medical College, Huazhong University of Science and Technology, Wuhan, China

## Abstract

**Background:**

Osteoporosis (OP) is a systemic skeletal disease marked by bone mass reduction and bone tissue destruction. Hormone replacement therapy is an effective treatment for post-menopausal OP, but estrogen has poor tissue selectivity and severe side effects.

**Results:**

In this study, we constructed a poly(lactic-co-glycolic acid) (PLGA) nanoparticles (NPs)-based drug delivery system to co-load 17β estradiol (E_2_) and iron oxide (Fe_3_O_4_) together, modified with alendronate (AL) to achieve bone targeting and realize a magnetically remote-controllable drug release. The NPs were fabricated through the emulsion solvent diffusion method. The particle size was approximately 200 nm while the encapsulation efficiency of E_2_ was 58.34 ± 9.21%. The NPs were found to be spherical with a homogenous distribution of particle size. The NPs showed good stability, good biocompatibility, high encapsulation ability of E_2_ and excellent magnetic properties. The NPs could be effectively taken up by Raw 264.7 cells and were effective in enriching drugs in bone tissue. The co-loaded NPs exposed to an external magnetic field ameliorated OVX-induced bone loss through increased BV/TV, decreased Tb.N and Tb.Sp, improved bone strength, increased PINP and OC, and downregulated CTX and TRAP-5b. The haematological index and histopathological analyses displayed the NPs had less side effects on non-skeletal tissues.

**Conclusions:**

This study presented a remote-controlled release system based on bone-targeted multifunctional NPs and a new potential approach to bone-targeted therapy of OP.

**Graphic abstract:**

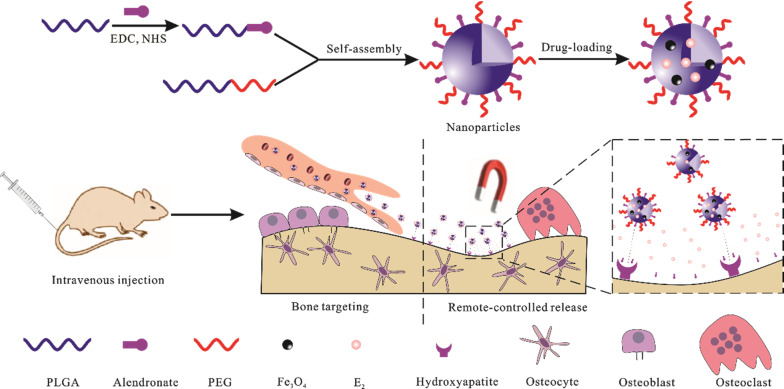

**Supplementary Information:**

The online version contains supplementary material available at 10.1186/s12951-021-00976-4.

## Introduction

Osteoporosis (OP) is a systemic skeletal disease marked by bone mass reduction and bone tissue destruction, causing skeletal fragility and high fracture risk, which is especially common in elderly and post-menopausal women [1]. By 2050, the proportion of osteoporotic fractures in all fractures will be risen to 50%, and the calculable cost of osteoporotic hip fractures will come up to $131 billion around the world [2]. With the continuing increase in the aging population, OP is becoming a global public health concern, seriously endangering human health, and research focusing on anti-OP is much warranted. Knowledge of bone biology has progressed dramatically over the past 30 years. Meanwhile, numerous therapeutic drugs, including estrogen, calcitonin, bisphosphonates, raloxifene and RANK ligand inhibitors, have come onto the market. However, their clinical applications are severely restricted by their side effects and high costs. Moreover, these drugs are generally administered systematically and the high dose and frequent administration necessary due to their low bone-targeting efficiency can lead to serious side effects [3]. The non-skeletal in vivo bio-distribution of these therapeutic agents causes unexpected side-effects on non-skeletal tissues and organs, while low concentrations of therapeutic drugs in bone reduces their clinical efficacy [4, 5]. The above limitations bring enormous challenges to overcome in hodiernal anti-OP strategies. Consequently, the progress of an effectively innovative bone-targeting delivery system is urgently needed [6, 7].

The skeleton is an unusual tissue in that a great deal of hormones are desired to regulate its growth, maturation, and remodeling, with estrogen as the most important one. During the period of menopause, women often exhibit rapid bone loss accompanied by an elevated risk of OP and related fractures, resulting from the decline in endogenous estrogen production [8]. The phenomenon of idiopathic OP occurring in the perimenopause, also called postmenopausal OP (PMOP). The relationship between 17β-estradiol (E_2_) and bone biology was confirmed [9]. After that, estrogen therapy solely or combined with hormone therapy (HT) became one of the most efficient anti-OP therapies until 1995 when Fosamax, an alendronate sodium, was approved [10]. It has been well documented that estrogen replacement therapy (ERT) prevents bone loss, as well as averting enhances in bone resorption and secondary hyperparathyroidism [11] and equivalent to approximately 70% of OP-related fractures [12, 13]. However, the Women’s Health Initiative (WHI) showed that HT enhanced health risks and contributed to heart disease, which caused doubt and started a societal transition out of use HT in anti-OP [10, 14, 15]. Subsequently ERT, once the primary choice for PMOP, was reported to be related to an enhancive risk of cancer and cardiovascular disease in postmenopausal women. The American College of Physicians (ACP) recommended against the clinic application of HT (recommendation 5) in their 2017 clinical guidelines regarding OP management [16]. Of note, the WHI carried out a study on postmenopausal women aged from 50 to 79 to investigate the effects of HT on the prevention of cardiovascular disease [14]. This recommendation of the ACP raised concern and has been extensively questioned and debated [10, 17, 18].

Despite this controversy, the effectiveness of estrogen-based HT on bone protection has been proven. It has been well established that the critical factor in maintaining bone homeostasis is estrogen receptors (ERs) and their signaling pathways. Accumulating evidence supporting the use of HT may be regarded as a choice of primary treatment for OP in women during early menopausal period [9, 14]. To circumvent the side effects caused by ERT, selective ER modulators (SERMs), synthetic nonsteroidal agents exerting both estrogen agonistic and antagonistic properties in different tissues, were introduced [19]. SERMs act as agonists by varying the activity of osteoclasts and osteoblasts while causing fewer side effects than estrogen. Neither raloxifene, bazedoxifene, nor lasofoxifene have shown an increased occurrence rate of endometrial hyperplasia or carcinoma. However, all SERMs have been resulted in increased hot flushes and venous thromboembolic events. The role of estrogen in maintaining bone health in both females and males is well recognized [20]. However, the drawbacks of estrogenic drugs (estrogen or SERMs) have limited the clinical application of hormone replacement therapy (HRT). It would be highly valuable to develop novel estrogenic substances for purpose of preventing bone loss and reducing adverse effects. Estrogenic drugs physiologically targeted to bone tissue may have decreased side effects and increased efficacy and safety.

In this study, we successfully constructed a stimulus responsive nanoparticles (NPs) based on poly(lactic-co-glycolic acid) (PLGA). Firstly, PLGA were modified with alendronate (AL) via chemical bonds for bone-targeting. Secondly, the sex steroid hormones (E_2_) and Fe_3_O_4_ magnetic NPs were encapsulated into PLGA-based matrices. It is well known that PLGA and Fe_3_O_4_ NPs have been approved to put into clinical apply by FDA due to their good biocompatibility [21]. Base on the Brownian motion and Néel relaxation of magnetic NPs, the Fe_3_O_4_ NPs can function as “heat generators” to achieve a remote-controllable drug release [22]. The goal of this drug delivery system is to achieve a magnetically remote-controllable drug release under the guidance of an external magnetic field. We investigated the characteristics, stability and biocompatibility of the constructed NPs. The resultant in vitro drug release, cellular uptake capacity and targeting effect was also investigated. In the next moment, we used a three-month study in an ovariectomized (OVX) rat model to explore the effect and role of NPs in anti-OP. Our study provides a new strategy for bone-targeting drug delivery against OP.

## Materials and methods

### Materials

PLGA (Resomer® RG 503 H, 50:50, Mw 24,000–38,000), AL, N-(3-dimethylaminopropyl)-N'-ethylcarbodiimide hydrochloride (EDC), N-hydroxysuccinimide (NHS) and 3-(4,5-dimethyl-thiazol-2-yl)-2,5-diphenyl tetrazolium bromide (MTT) were purchased from Sigma-Aldrich (St. Louis, MO, USA). Poly(ethylene glycol) methyl ether-block-poly(lactide-co-glycolide) (mPEG 500, 2000, 5000-PLGA 50:50 18000) was produced by Jinan Daigang Biomaterial Co., Ltd (Jinan, China). Poly(vinyl alcohol) (PVA) (Mowiol® 4–88) was obtained from Kuraray Specialities Europe GmbH (Frankfurt, Germany). E_2_ was obtained from Selleck Chemicals (Houston, TX, USA) and 1,1-dioctadecyl-3,3,3,3-tetramethylindotricarbocyanine iodide (DiR iodide) was produced by AAT Bioquest (Sunnyvale, CA, USA). Dulbecco’s modified Eagle medium (DMEM) and fetal bovine serum (FBS) were supplied by Gibco BRL (Carlsbad, CA, USA). Penicillin–streptomycin and trypsin without EDTA were ordered from Hyclone (Logan, UT, USA). Coumarin 6 (C6) and Hoechst 33342 were purchased from Beyotime Institute of Biotechnology (Haimen, China). Millipore ultrapure water with a resistivity of 18.2 MΩ.cm was used. All the solvents of analytical grade were ordered from Sinopharm Chemical Reagent Co., Ltd (Shanghai, China).

### Cells and animals

The murine macrophage cell line (Raw 264.7) and human umbilical vein endothelial cells (HUVECs) were purchased from the American type culture collection (ATCC). All cells were cultured in DMEM containing 10% FBS, 100 U/mL penicillin and 100 μg/mL streptomycin in a humidified atmosphere incubator with 5% CO_2_ at 37 °C.

Specific pathogen-free (SPF) female Sprague–Dawley (SD) rats (230–250 g, 12 weeks old) were purchased from Beijing Vital River Laboratory Animal Technology Co., Ltd. (Certificate No. SCXK 2016-0006) and raised in the Laboratory Animal Center, Huazhong University of Science and Technology (Certificate No. SCXK 2016–0057). The rats were housed at room temperature (25 °C) in 60 ± 10% humidity under the natural light–dark cycle throughout the experiments. All animal experiments were carried out according to Chinese law and were approved by the local Ethical Committee.

### Synthesis of AL-PLGA

The AL-PLGA was synthesized as reported previously with slight modification [23]. In brief, PLGA (140 mg, 0.0046 mmol) was dissolved in 4 mL dichloromethane (DCM). Then EDC (8.82 mg, 0.046 mmol) and NHS (5.30 mg, 0.046 mmol) were added and stirred at room temperature for 1 h to obtain NHS-activated PLGA. Next, AL (1.50 mg, 0.0046 mmol) was added to this solution and stirred overnight. Then DMSO was added to the mixture. The solvent was partly removed by volatilization while the remainder was dialyzed against water, then subjected to freeze drying. The synthesis of AL-PLGA was certified by ^1^H-NMR (600 MHz, DMSO-d6) (Ascend™ 600 MHz, Bruker, Germany).

### Preparation of NPs

PEG-PLGA-AL@Fe_3_O_4_/E_2_ NPs were prepared through the emulsion solvent diffusion method [24]. Briefly, mPEG-PLGA (12.5 mg) and AL-PLGA (12.5 mg) were dissolved in ethyl acetate (1.25 mL) containing Fe_3_O_4_ NPs and E_2_. Then the solution was added dropwise into 2% PVA aqueous solution (1.25 mL), stirred for 1 h and ultrasonically emulsified at 500 J for 30 s (HN-1000Y, Hanuo Instruments, Shanghai, China). In order to allow the organic phase diffusing into the aqueous phase, the solution was added into ultrapure water. After the evaporation of ethyl acetate, PEG-PLGA-AL@Fe_3_O_4_/E_2_ NPs were purified using a magnetic-activated cell sorting system (MACS). Fe_3_O_4_-unloaded PLGA NPs (PLGA@E_2_ NPs, AL-PLGA@E_2_ NPs, PEG-PLGA@E_2_ NPs and PEG-PLGA-AL@E_2_ NPs) were prepared following a similar protocol while purification was performed with a disposable ultrafiltration device (Vivaspin® 20, Sartorius AG, Goettingen, Germany).

### Characterization of NPs

The hydrodynamic diameter, polydispersity index (PDI) and zeta potential of PEG-PLGA-AL@Fe_3_O_4_/E_2_ NPs suspended in ultrapure water were measured by Dynamic light scattering (DLS) (Nano-ZS90, Malvern Panalytical, Malvern, UK). The morphology of prepared PEG-PLGA-AL@Fe_3_O_4_/E_2_ NPs was visualized by scanning electron microscopy (SEM) (MIRA3, Tescan, Czech Republic), transmission electron microscopy (TEM) (H-7000FA, Hitachi, Tokyo, Japan) and atomic force microscopy (AFM) (SPA-400, Seiko Instruments Inc., Chiba, Japan). The magnetic properties of PEG-PLGA-AL@Fe_3_O_4_/E_2_ NPs were observed by magnetic force microscopy (MFM) (Dimension Edge, Bruker, Germany).

The long-term stability of PEG-PLGA-AL@Fe_3_O_4_/E_2_ NPs in 0.01 M phosphate buffered saline (PBS) was evaluated by DLS for 15 days [25]. The serum stability test was carried out as reported previously. Briefly, the prepared NPs were dispersed in FBS at a concentration of 1 mg/mL and the absorbance values of 560 nm were measured after 4 h.

### Drug encapsulation and release study

In order to test encapsulation efficiency (EE) (Eq. 1) and drug loading efficiency (DLE) (Eq. 2) of E_2_, the prepared PEG-PLGA-AL@Fe_3_O_4_/E_2_ NPs were centrifuged at 14,000 rpm for 10 min and the precipitate was resuspended in acetonitrile. Thereafter the filtrate was collected through a 0.22 μm filter membrane and analyzed. With regard to Fe_3_O_4_, the NP suspension was digested with nitric acid–perchloric acid and redissolved in water for subsequent analysis.

1 $${\text{ EE}}\% = \frac{{{\text{amount}\;\text{of}}\; {\text{E}}_{2}\, \left( {{\text{or}}\; {\text{Fe}}_{3} {\text{O}}_{4} } \right)\; {\text{in}}\; {\text{NPs}}}}{{{\text{total}\; \text{amount}}\, {\text{of}}\; {\text{E}}_{2} \left( {{\text{or}}\; {\text{Fe}}_{3} {\text{O}}_{4} } \right)}} \times 100\%$$

2 $${\text{DLE}}\% = \frac{{{\text{amount}}\;{\text{of}}\;{{\text{E}}_2}\left( {{\text{or}}\;{{\text{Fe}}_3}{{\text{O}}_4}} \right)\;{\text{in}}\;{\text{NPs}}}}{{{\text{amount}}\;{\text{of}}\;{{\text{E}}_2}\left( {{\text{or}}\;{{\text{Fe}}_3}{{\text{O}}_4}} \right){\text{in}}\;{\text{NPs}} + {\text{amount}}\;{\text{of}}\;{\text{polymer}}\;{\text{used}}}} \times 100\%$$

To evaluate the release behavior of E_2_, the PEG-PLGA-AL@Fe_3_O_4_/E_2_ NPs were kept in a temperature-controlled release medium using a dialysis method. The experiments were carried out at 25, 37 and 45 °C. The NPs were dispersed into 10 mL 0.01 M PBS at pH 7.4 in a water bath shaker at 60 rpm. At predetermined intervals, the medium was completely removed followed by addition of 10 mL fresh PBS. The amount of released E_2_ was determined by high-performance liquid chromatography (HPLC). Furthermore, TEM and SEM analyses were used to study possible morphological changes of the particles when subjected to external heat.

### Cellular uptake assay

The cellular uptake capability of NPs was evaluated by confocal laser scanning microscopy (CLSM) and flow cytometry (FCM). For flow cytometry, C6-labeled NPs (PLGA@C6 NPs, AL-PLGA@C6 NPs, PEG-PLGA@C6 NPs, PEG-PLGA-AL@C6 NPs and PEG-PLGA-AL@Fe_3_O_4_/C6 NPs) were incubated with Raw 264.7 cells. After washing with PBS to eliminate unbound NPs, the cells were trypsinized for subsequent analysis.

For qualitative assay by CLSM, the cells were fixed with 4% paraformaldehyde after incubation with C6-labeled NPs. Then the cell nuclei were stained with Hoechst 33342 followed by imaging under CLSM.

### Cytotoxicity study

The cytotoxicity study was conducted using the MTT assay. In brief, HUVECs were incubated with different concentrations of NPs (PLGA NPs, AL-PLGA NPs, PEG-PLGA NPs, PEG-PLGA-AL NPs and PEG-PLGA-AL@Fe_3_O_4_ NPs) for 24 h. Then MTT was added to the mixture for an extra 4 h, the precipitate was redispersed with DMSO, and the absorbance values at 570 nm were recorded. The cell survival rate was calculated using Eq. 3, where As and Ac represented the absorbance values of the sample and the untreated control group, respectively.

3 $${\text{Cell}}\; {\text{viability}}\% = \frac{{{\text{As}}}}{{{\text{Ac}}}} \times 100\%$$

### Hemolysis assay

The hemolysis assay was carried out according to a previous study [26]. Briefly, whole blood from female SD rats was collected in tubes containing heparin sodium. The blood was centrifuged at 3000 rpm for 10 min at 4 °C, and fresh red blood cells (RBCs) were washed three times in cold normal saline (NS). Then the PEG-PLGA-AL@Fe_3_O_4_/E_2_ NPs were incubated with diluted RBCs (2%) at 37 °C for 2 h followed by centrifugation at 3000 rpm for 10 min. The absorbance values of the supernatant at 540 nm were measured using a microplate reader (EnSpire, PerkinElmer, Waltham, MA, USA). The NS and ultrapure water were taken as the negative control and positive control, respectively. The hemolysis ratio was calculated using Eq. 4, where As, Anc and Apc represent the absorbance values of the sample, negative control and positive control separately.

4 $${\text{ Hemolysis ratio}}\% = \frac{{{\text{As}} - {\text{Anc}}}}{{{\text{Apc}} - {\text{Anc}}}} \times 100\%$$

### In Vivo biodistribution study

DiR-labeled NPs (PLGA@DiR NPs, AL-PLGA@DiR NPs, PEG-PLGA@DiR NPs, PEG-PLGA-AL@DiR NPs and PEG-PLGA-AL@Fe_3_O_4_/DiR NPs) were prepared following a similar protocol, with DiR added into the organic phase before emulsification. SD rats were administered DiR-labeled NPs at a dose of 12.5 mg/kg via tail intravenous injection. After 24 h, the rats were examined using an in vivo imaging system (IVIS, Ex 750 nm, Em 790 nm) (In-Vivo Fx Pro, Bruker, Germany). Thereafter, the rats were sacrificed by administration of excess pentobarbital sodium and the heart, liver, spleen, lung, kidney and bones (lumbar vertebra, femur, tibia and fibula) were separated for subsequent analysis. After washed three times with saline, fluorescent quantitative analysis of distribution in excised bone tissue were implemented through the imaging system.

### Therapeutic assay of osteoporosis in vivo

The therapeutic assay was carried out in SD rats and OVX rats were used to create a PMOP model. The rats were operated and divided into different groups randomly at 4 weeks before NP treatment (nine rats per group). The rats in each group were subjected to nine periodic tail intravenous injections of NPs with an E_2_ dose of 63 μg/kg once a week [27]. Serum and whole blood were collected for analysis at the end of treatment. After euthanizing the rats with excess pentobarbital sodium, the wet weight of the uterus and the body weight were measured and the right tibia was collected for subsequent hematoxylin–eosin (H&E) staining. Sections of the main organs (liver, uterus and small intestine) were obtained and stained with H&E. In addition, the right femur was removed and scanned using a Micro-CT system (Skyscan 1176, MicroCT, Kontich, Belgium). The bone volume (BV/TV), trabecular thickness (Tb.Th), trabecular number (Tb.N) and trabecular separation (Tb.Sp) were calculated from the three-dimensional CT reconstruction.

### Three-Point bending test of the femur

We investigated the biomechanical properties of the right femur in OVX rats using a previously-validated three-point bending test. All the bones were stripped of surrounding muscle tissue and stored in 0.9% NaCl in a 4 °C refrigerator. Each sample was subjected to a three-point bending test to failure using a universal material testing machine (Autograph AG-5000A; Shimadzu Corp., Kyoto, Japan). The distance between the two lower supports (span) was 25 mm. A metal pole was centered between the supports and the loading speed of the metaphysis was 5.0 mm/min for all tests. On the basis of the load-deformation curve, we recorded the following biomechanical properties: maximum load (N), maximum strength (MPa) and elastic modulus (10^3^ GPa).

### Fluorescent double-labeling with tetracycline and calcein

In order to assess bone formation, rats received intraperitoneal injection of 20 mg/kg calcein and 30 mg/kg of tetracycline hydrochloride, respectively, on the 10th and 4th day prior to euthanasia. Briefly, the proximal part of the right tibia was fixed in 70% ethanol at 4 °C, and then dehydrated with 70, 80, 85, 90, 95, 100 and 100% ethanol for 3 days each. After dehydration, the specimens were embedded in 4% methyl methacrylate. The bone specimens were then cut into 30-µm sections and observed by fluorescence microscopy. The mineral apposition rate (MAR) was calculated based on the distance between the two fluorescently-labeled lines (Eq. 5).

5 $${\text{MAR}}\left( {{{\upmu}} {\text{m}}/{\text{day}}} \right) = \frac{{{\text{double} \;\text{fluorescence}\; \text{line}\;\text{spacing}}}}{{{\text{days} \;\text{between} \;\text{the} \;\text{two} \;\text{lines}}}} \times 100\%$$

### Hematological parameter determination

Blood samples were obtained from rats using EDTA as anticoagulant. White blood cell (WBC), red blood cell (RBC), hemoglobin (HGB) and blood platelet (PLT) were measured using a hematology autoanalyzer. We also detected the serum alanine aminotransferase (ALT), aspartate aminotransferase (AST), alkaline phosphatase (ALP), creatinine (Cr) and total cholesterol (TC).

### Detection of bone turnover markers in serum

Collected blood samples were centrifuged for 10 min to extract serum. The serum levels of calcium (Ca) or phosphorus (P) were analyzed using an atomic absorption spectrometer. The content of bone turnover markers, such as tartrate-resistant acid phosphatase 5b (TRAP-5b), osteocalcin (OC), N-terminal propeptide of type-I procollagen (PINP) and C-telopeptide (CTX) in the serum was examined by enzyme-linked immunosorbent assay (ELISA) according to the protocols provided by the manufacturer (Elabscience Biotechnology, Bethesda, MD, USA).

### Statistical analysis

Experiments were performed three times. Data are plotted as mean values ± standard deviation. Student’s t test was performed to compare values between two groups while Tukey’s multiple comparisons test was used for data analysis with more than one comparison. A statistically-significant difference was defined as *P* < 0.05.

## Results

### Synthesis and characterization of AL-PLGA

AL-PLGA was synthesized via a two-step process as shown in Additional file 1: Figure S1a. Acid-terminated PLGA was activated by EDC/NHS and subsequently bound to AL containing an amino group. The basic chemical structure of AL-PLGA was confirmed by ^1^H-NMR. The signals at 1.49, 4.92 and 5.22 ppm in the ^1^H-NMR spectrum of PLGA (Additional file 1: Figure S1b), correspond to the methyl groups of lactic acid, the methylene groups of glycolic acid and the methine groups of lactic acid, respectively.

Compared to the PLGA spectrum, ^1^H-NMR characterization of AL-PLGA (Additional file 1: Figure S1c), contained signals at 1.59 ppm (–CCH_2_CH_2_CH_2_NHCO–) and 2.91 ppm (–CCH_2_CH_2_CH_2_NHCO–). The presence of ALN was verified by a small peak at 1.59 ppm, which is in line with data previously reported [28, 29]. Thus the ^1^H-NMR spectrum indicated the successful synthesis of AL-PLGA.

### Preparation and characterization of NPs

#### Particle size, surface charge and morphology of NPs

**Fig. 1:**
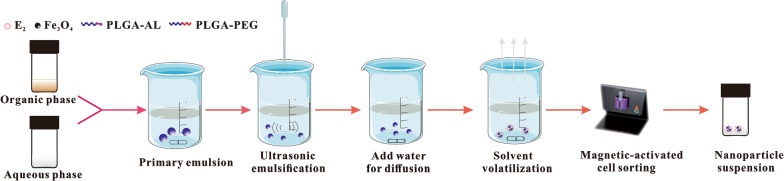
Schematic illustration of the preparation of NPs

**Fig. 2:**
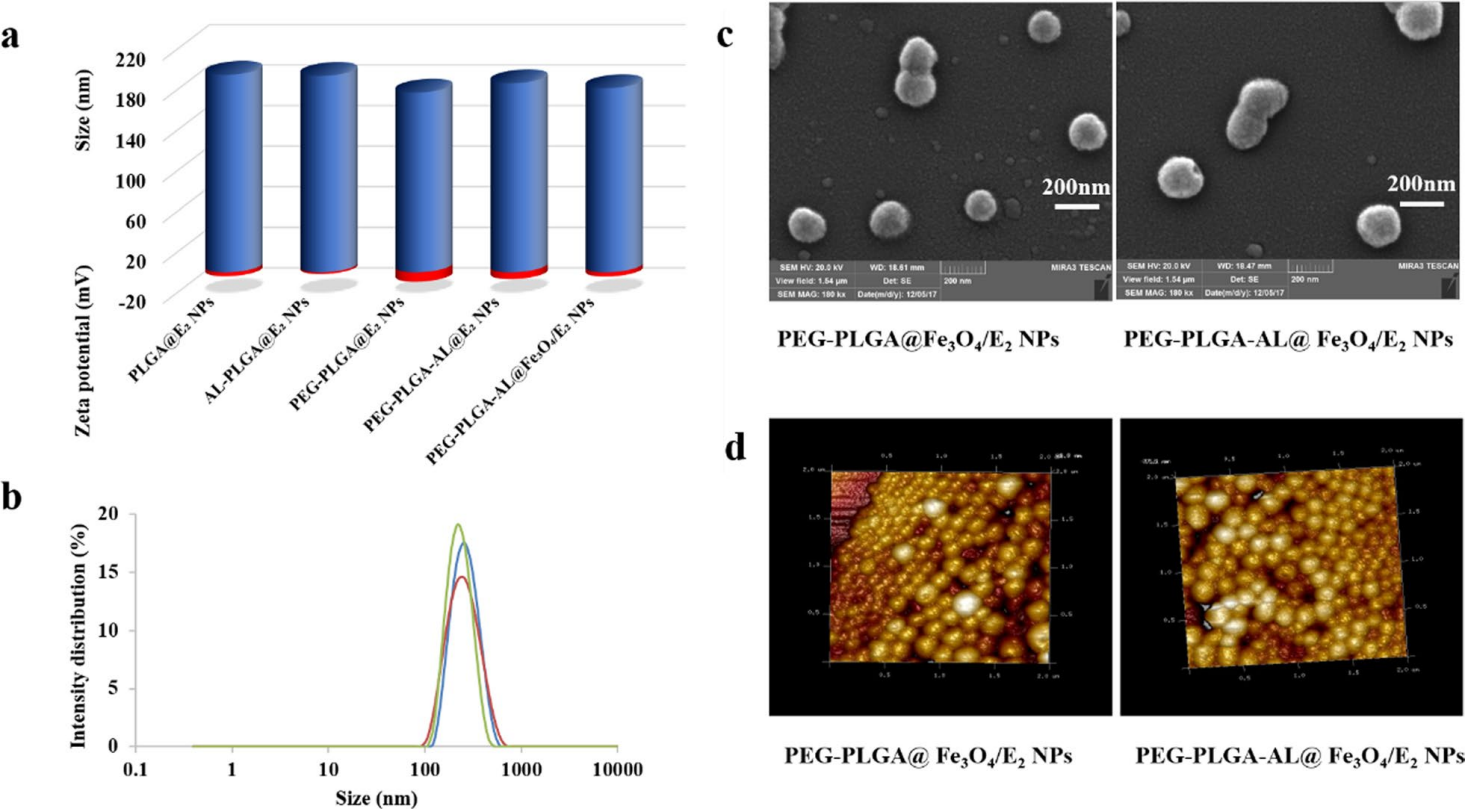
General characteristics of NPs. **a** Hydrodynamic diameter (blue) and zeta potential (red) of NPs by DLS. **b** Hydrodynamic diameter distribution of PEG-PLGA-AL@Fe_3_O_4_/E_2_ NPs at different measurement time: 24 h (red line), 48 h (blue line), 72 h (green line). **c** SEM images and **d** AFM images of PEG-PLGA@Fe_3_O_4_/E_2_ NPs and PEG-PLGA-AL@Fe_3_O_4_/E_2_ NPs

As shown in Fig. 1, the emulsion solvent diffusion volatilization method was used to prepare E_2_ and iron oxide (Fe_3_O_4_) co-loaded NPs. DLS of prepared PEG-PLGA-AL@Fe_3_O_4_/E_2_ NPs revealed a mean size (hydrodynamic diameter) of 181.4 ± 1.2 nm, a PDI of 0.236 ± 0.008 and a zeta potential of − 4.18 ± 0.78 mV (Fig. 2a). In comparison, the results of PLGA NPs without Fe_3_O_4_ (PLGA@E_2_ NPs, AL-PLGA@E_2_ NPs, PEG-PLGA@E_2_ NPs and PEG-PLGA-AL@E_2_ NPs) did not differ substantially, with a particle size of slightly lower than 200 nm (Fig. 2b). The morphology of E_2_ and Fe_3_O_4_ co-loaded NPs was visualized by SEM, TEM and AFM. As observed in Fig. 2c, d, the NPs displayed a spherical shape and a homogenous distribution, and the AL modification had no significant influence on the morphology of NPs. The particle size observed by SEM had a good correlation with the value measured by particle size analysis.

#### Magnetic properties of NPs

**Fig. 3:**
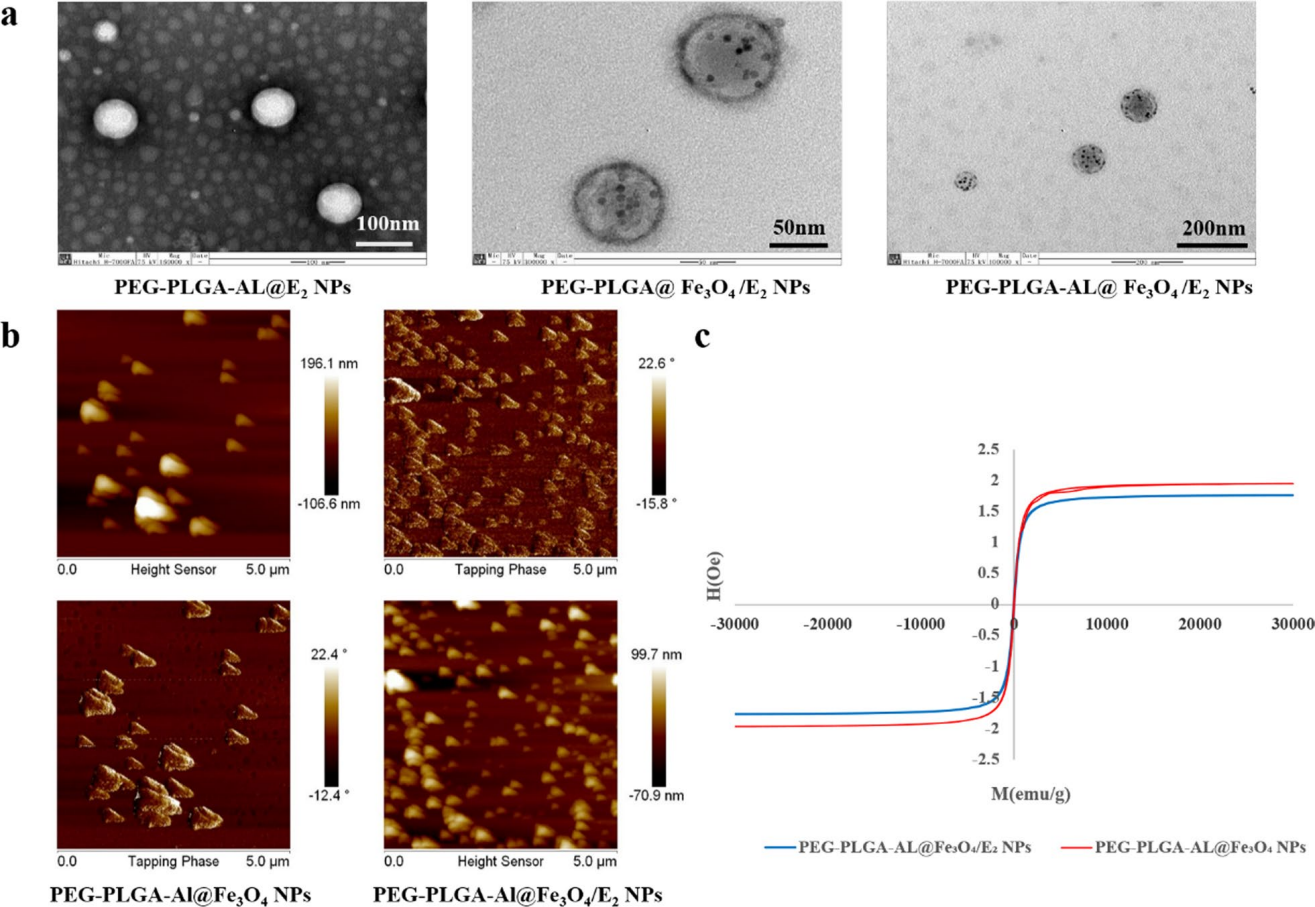
Magnetic properties of NPs. TEM images (**a**) of PEG-PLGA-AL@E_2_ NPs, PEG-PLGA@Fe_3_O_4_/E_2_ NPs and PEG-PLGA-AL@Fe_3_O_4_/E_2_ NPs. MFM images (**b**) and magnetic hysteresis curves (**c**) of PEG-PLGA-AL@Fe_3_O_4_ NPs and PEG-PLGA-AL@Fe_3_O_4_/E_2_ NPs

The magnetic properties observed by TEM (Fig. 3a) revealed that the Fe_3_O_4_ NPs were distributed homogenously within the polymeric matrices. Furthermore, we investigated the magnetic domain in PEG-PLGA-AL@Fe_3_O_4_/E_2_ NPs by MFM. There was a relatively good correlation in terms of the color observed in an MFM image and measurement from magnetism analysis (Fig. 3b). As seen in Fig. 3c, the saturation magnetization of PEG-PLGA-AL@Fe_3_O_4_ NPs and PEG-PLGA-AL@Fe_3_O_4_/E_2_ NPs were similar, with a value of around 2 emu/g. Besides, the remanence and coercive force were both close to zero. The PEG-PLGA-AL@Fe_3_O_4_/E_2_ NPs were superparamagnetic, which could be utilized to promote drug release by magnetic fluid hyperthermia. These results also indicated that Fe_3_O_4_/E_2_-loaded NPs (PEG-PLGA-AL@Fe_3_O_4_/E_2_ NPs) have a strong negative surface charge and superparamagnetic property for stabilizing the drug-loaded NPs.

**Fig. 4:**
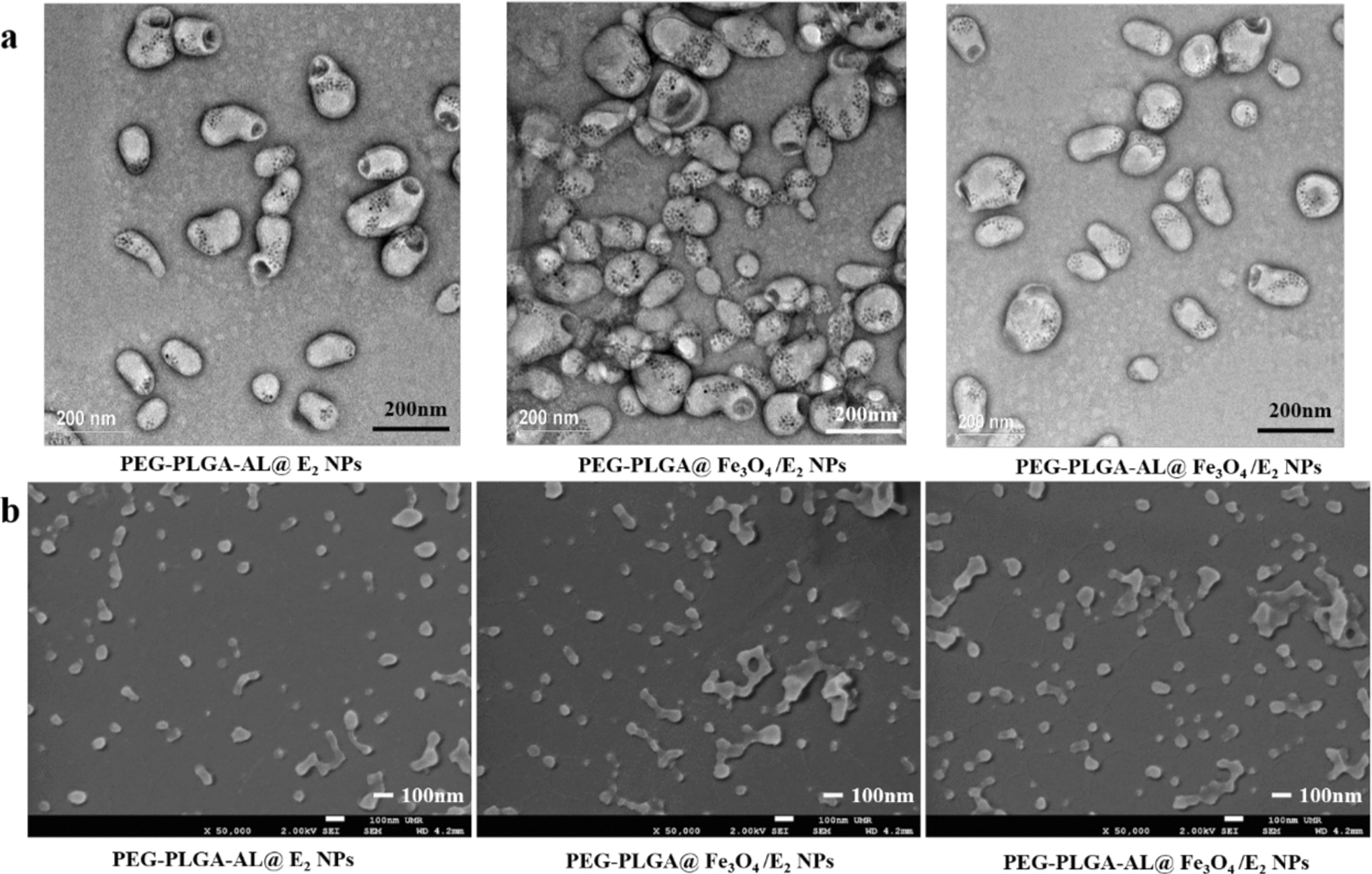
TEM (**a**) and SEM (**b**) images of inductively-heated PEG-PLGA-AL@E_2_ NPs, PEG-PLGA@Fe_3_O_4_/E_2_ NPs and PEG-PLGA-AL@Fe_3_O_4_/E_2_ NPs up to 45 °C. Experiments were carried out using a frequency of 170 kHz

Next, TEM and SEM analyses were used to observe the effect of external heating on particle morphology. As seen in Fig. 4a, most of the NPs showed a visible variation in morphology from a spherical shape to an amorphous shape. Furthermore, some NPs were fragmented and aggregated, as though “broken”. However, SEM images of PEG-PLGA-AL@E_2_ NPs, PEG-PLGA@Fe_3_O_4_/E_2_ NPs and PEG-PLGA-AL@Fe_3_O_4_/E_2_ NPs when subjected to external heating at 45 °C for about two days revealed no perceptible changes in the morphology of the NPs (Fig. 4b). These results were consistent with our previous research [24].

#### Stability of NPs

**Fig. 5:**
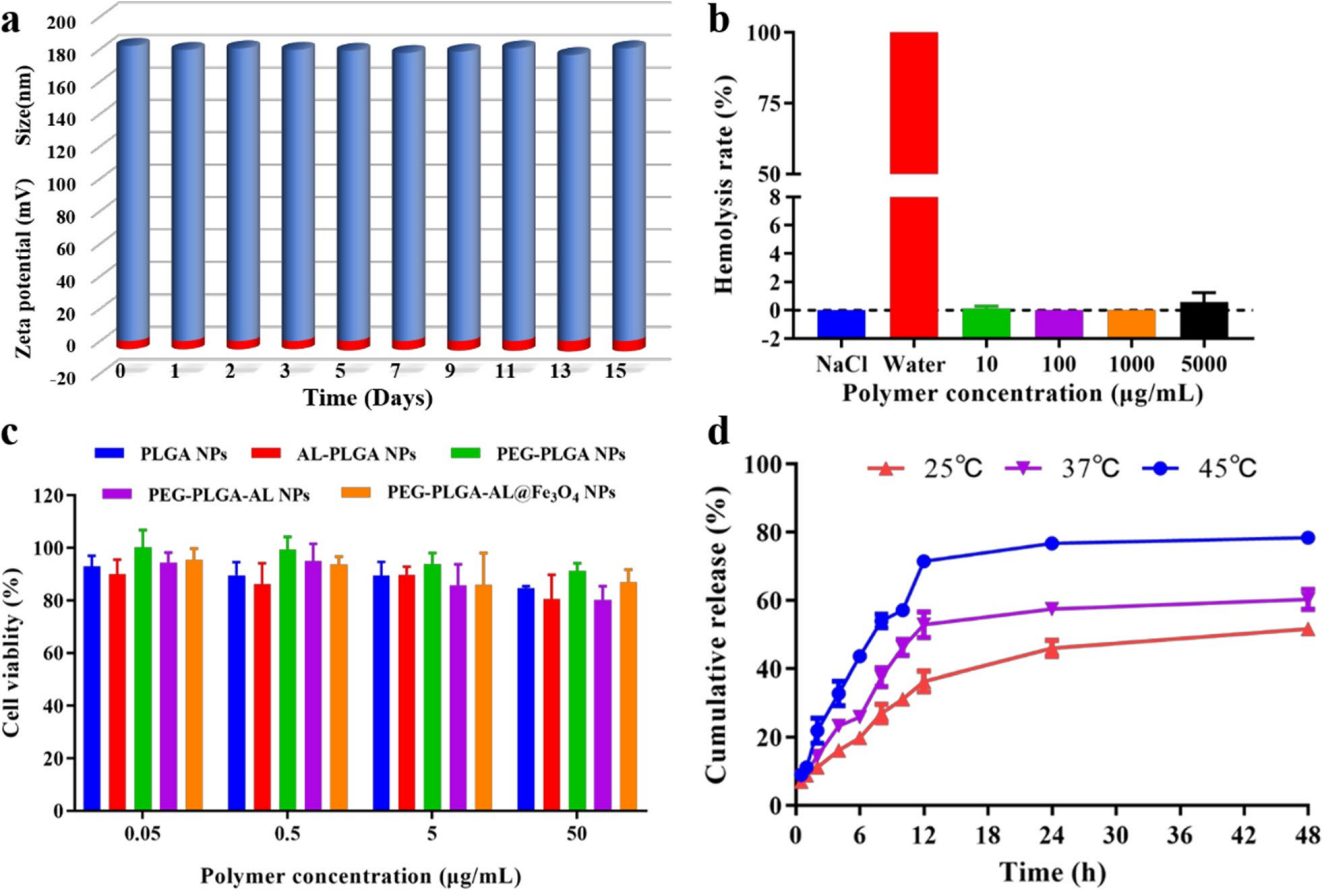
Characterization of NPs. **a** Hydrodynamic diameter (blue) and zeta potential (red) of different NPs over 15 days. **b** The hemolysis ratio of PEG-PLGA-AL@Fe_3_O_4_/E_2_ NPs of different concentrations incubated with RBCs. **c** In vitro cytotoxicity of NPs against HUVECs after treatment for 24 h (n = 3). **d** In vitro release profile of E_2_ from PEG-PLGA-AL@Fe_3_O_4_/E_2_ induced by external heating at 25 °C, 37 °C and 45 °C. The data are presented as mean ± SD (n = 3)

For sake of exploring the biomedical applications of NPs, the stability of different NPs was assessed by re-dispersing NPs in FBS and pH 7.4 PBS. Firstly, we continuously monitored the particle size and the surface zeta potential of PEG-PLGA@Fe_3_O_4_/E_2_ NPs for 15 days in PBS using DLS. As shown in Fig. 5a, the hydrodynamic diameter of PEG-PLGA@Fe_3_O_4_/E_2_ NPs varied slightly from 181.90 ± 0.24 to 180.40 ± 0.24 nm, while the zeta potential decreased from − 4.96 ± 0.51 to − 6.35 ± 1.97 mV, but no remarkable change was perceived within 15 days. Furthermore, the absorbance of PEG-PLGA@Fe_3_O_4_/E_2_ NPs at 560 nm was investigated over 4 h in PBS and FBS. As shown in Additional file 1: Figure S2, the size of NPs remained basically stable with negligible fluctuation for more than 72 h both in PBS and FBS.

#### Biocompatibility of NPs

To investigate the biocompatibility of the NPs, we adopted the RBC hemolysis assay to quantify the properties of materials responsible for causing membrane damage. As shown in Fig. 5b, the hemolytic activities of PEG-PLGA-AL@Fe_3_O_4_ NPs were assessed for different concentrations within the range from 0.01 to 5 mg/mL. None of the groups showed visible hemolytic activities within the tested concentration range. The hemolytic percentages induced by all NPs were significantly less than 2% even at concentrations up to 5 mg/mL. Then the cytotoxicity of materials was quantified using the MTT assay. Generally, cell viability was more than 90% of the control group values over the whole concentration range (Fig. 5c). A low level of toxicity was found in cells treated with a high polymer concentration of 50 µg/mL. The results demonstrated that the NPs were appropriate for use over a wide safety range in drug delivery systems and suitable for intravenous administration. Collectively, these results showed that the NPs possessed good biocompatibility and had negligible cytotoxicity to normal cells.

### Drug encapsulation and release study

In order to assess the drug encapsulation, quantitative analysis of the EE and DLE of E_2_ in PLGA NPs was performed. The data in Additional file 1: Table S1 demonstrate the EE and DLE values of E_2_-loaded NPs. The EE of E_2_-loaded NP systems (PLGA@E_2_, AL-PLGA@E_2_, PEG-PLGA@E_2_ and PEG-PLGA-AL@E_2_) was 72.01 ± 2.61, 62.64 ± 7.13, 64.74 ± 16.55 and 58.31 ± 9.17%, respectively. Compared with PLGA@E_2_ NPs, the DLE of other E_2_-loaded NPs was slightly decreased. Increasing the E_2_ amount in PEG-PLGA-AL@Fe_3_O_4_/E_2_ NPs resulted in a higher loading efficiency of E_2_, while the DLE of NPs was not related to changes in the ratio of PLGA and Fe_3_O_4_ (Additional file 1: Table S2). Based on these results, 25:0.5 was chosen as the ratio of PLGA and Fe_3_O_4_. When the amount of Fe_3_O_4_ in PEG-PLGA-AL@Fe_3_O_4_/E_2_ NPs increased, the DLE of the Fe_3_O_4_ in NPs was slightly increased (Additional file 1: Table S3). When the ratio of PLGA and Fe_3_O_4_ increased from 25:0.25 to 25:0.75, keeping the E_2_ amount fixed, the EE of E_2_ decreased from 67.36 to 51.44% and the DLE from 1.30 to 1.00%. In order to explore the effect of Fe_3_O_4_ and E_2_, PEG-PLGA-AL@Fe_3_O_4_/E_2_ NPs should maintain a good entrapment efficiency for both Fe_3_O_4_ and E_2_, so 25:0.5 was selected as the optimal ratio of PLGA and Fe_3_O_4_ for further studies.

The in vitro release profiles of E_2_ from NPs under different conditions were measured by HPLC. As the results show in Fig. 5d, the release profile of E_2_ from PEG-PLGA-AL@Fe_3_O_4_/E_2_ exhibited an initial burst release during the first 6 h. The general tendency of the release profiles was consistent under different temperature conditions, with 46.01 ± 2.33, 57.55 ± 1.52 and 76.75 ± 1.59% of E_2_ released within 24 h when heated externally at 25, 37 and 45 °C, respectively. Afterwards, the E_2_ showed a slow and constant release, and approximately 50% of E_2_ remained un-released in the group at 25 °C even after 48 h. At 45 °C, PEG-PLGA-AL@Fe_3_O_4_/E_2_ showed an initial release of 54% within the first 8 h, and then reached a plateau of 71% after 12 h. There was no significant increase in drug release observed over the remaining 2 days.

### Cellular uptake capability of NPs

**Fig. 6:**
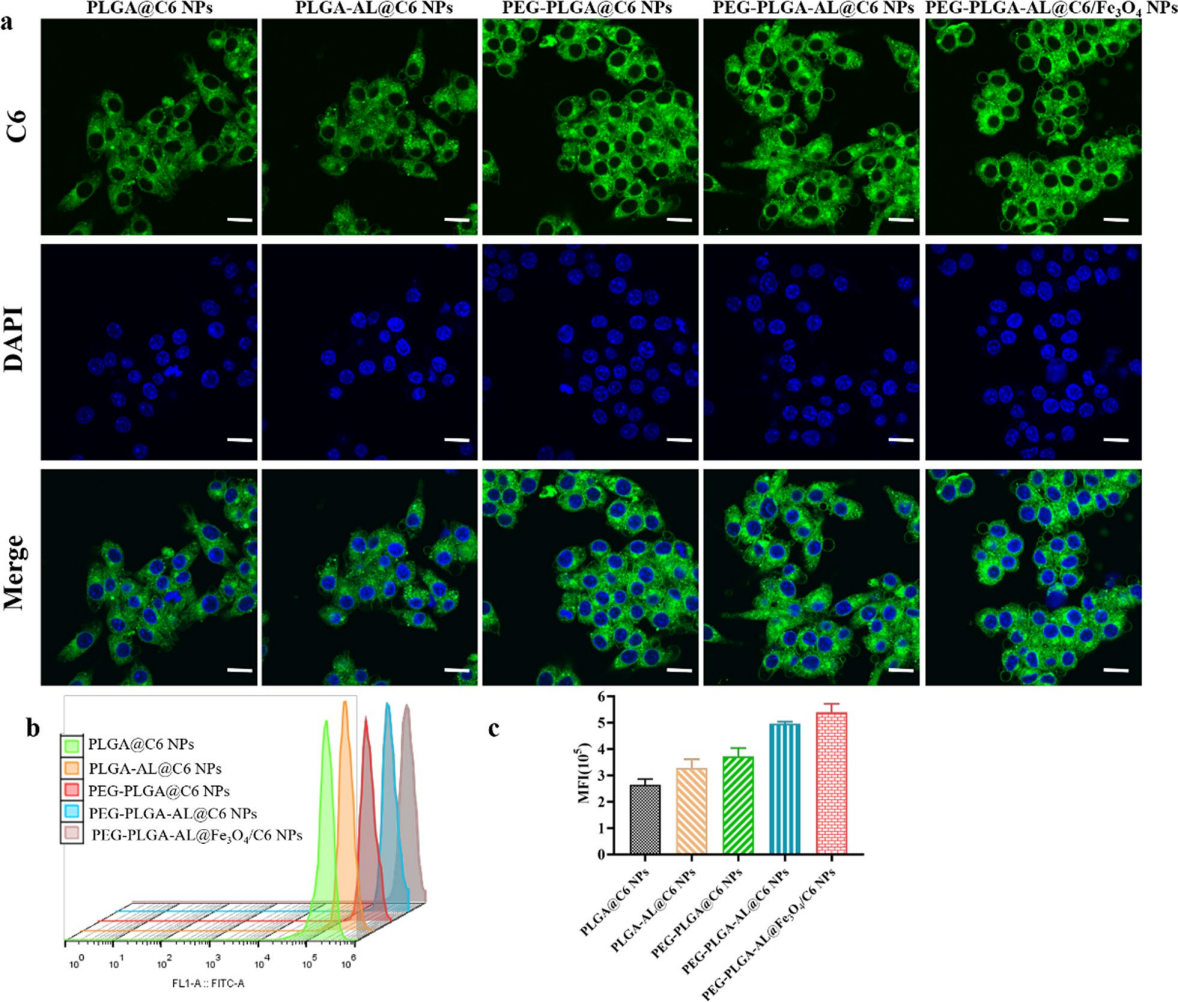
The cell uptake capability and cytotoxicity of NPs in Raw264.7 cells. CLSM images (**a**) and fluorescence intensity (**b**, **c**) of Raw 264.7 cells incubated with coumarin 6-loaded NPs (n = 3). Scale bar = 10 µm

The cellular uptake of different C6-loaded NPs by Raw 264.7 cells, a murine monocytic cell line which can differentiate into osteoclast-like cells under some circumstances, was qualitatively analyzed by confocal laser scanning microscopy. We adopted C6 (green) as the fluorescent label for tracking and comparison. As shown in Fig. 6a, all drug formulations resulted in fairly strong green fluorescence around the cytoplasm and closely accumulated around the nuclei (blue) of Raw 264.7 cells after 2 h of exposure. The results of cellular uptake capability were further confirmed through quantitative experiments through flow cytometry, as shown in Fig. 6b and c. The PEG-PLGA-AL@Fe_3_O_4_/C6 exhibited higher cell uptake capability when compared with that of other groups. The result was in a manner consistent with the result of previous qualitative experiments. This observation indicated that PEG-PLGA-AL@Fe_3_O_4_/C6 NPs were effectively taken up by Raw 264.7 cells.

### In vivo biodistribution study

**Fig. 7:**
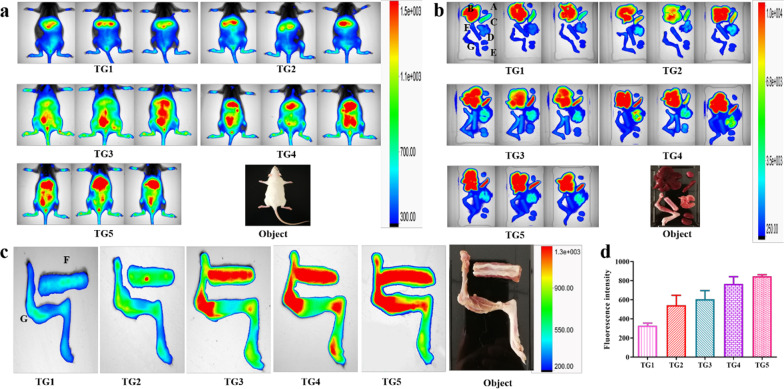
Bone-targeted ability of NPs. In vivo fluorescence images (**a**) of SD rats 24 h after tail vein injection of DiR-labeled NPs. Ex vivo fluorescence images (**b**) of excised organs at 24 h post injection: heart (A), liver (B), spleen (C), lung (D), kidney (E), lumbar vertebra (F), femur, tibia and fibula (G). Ex vivo fluorescence images (**c**) of lumbar vertebra (F), femur, tibia and fibula (G). Fluorescence intensity quantification (**d**) of excised bone tissue. Data are mean ± standard deviation (n = 3). Treatment groups TG1: PLGA@DiR NPs, TG2: AL-PLGA@DiR NPs, TG3: PEG-PLGA@DiR NPs, TG4: PEG-PLGA-AL@DiR NPs, TG5: PEG-PLGA-AL@Fe_3_O_4_/DiR NPs

To explore the bone-targeting ability of prepared NPs in vivo*,* DiR-loaded NPs (PLGA@DiR NPs, AL-PLGA@DiR NPs, PEG-PLGA@DiR NPs, PEG-PLGA-AL@DiR NPs and PEG-PLGA-AL@Fe_3_O_4_/DiR NPs) were intravenously injected into mice to investigate the fluorescence intensity in different organs. As shown in Fig. 7a, there was no obvious fluorescent signal at the injection site after administration with PLGA NPs. Meanwhile the fluorescent signal of PEG-PLGA-AL@Fe_3_O_4_/DiR NPs was much stronger than that of other groups, especially at 24 h after injection. Figure 7b shows that the fluorescent signal in liver and kidney was comparatively strong due to the large blood circulation and the accumulation of NPs. To study the biodistribution of NPs, the heart, liver, spleen, lung, kidney, and bones (lumbar vertebra, femur, tibia and fibula) were harvested at 24 h after injection. The PEG-PLGA-AL@Fe_3_O_4_/DiR accumulated mostly in the bone (Fig. 7c). Fluorescence intensity quantification (Fig. 7d) demonstrated that PEG-PLGA-AL@ Fe_3_O_4_/DiR NPs had a stronger fluorescent signal compared with PLGA@DiR NPs, AL-PLGA@DiR NPs or PEG-PLGA@DiR NPs.

### The safety of the NPs

**Fig. 8:**
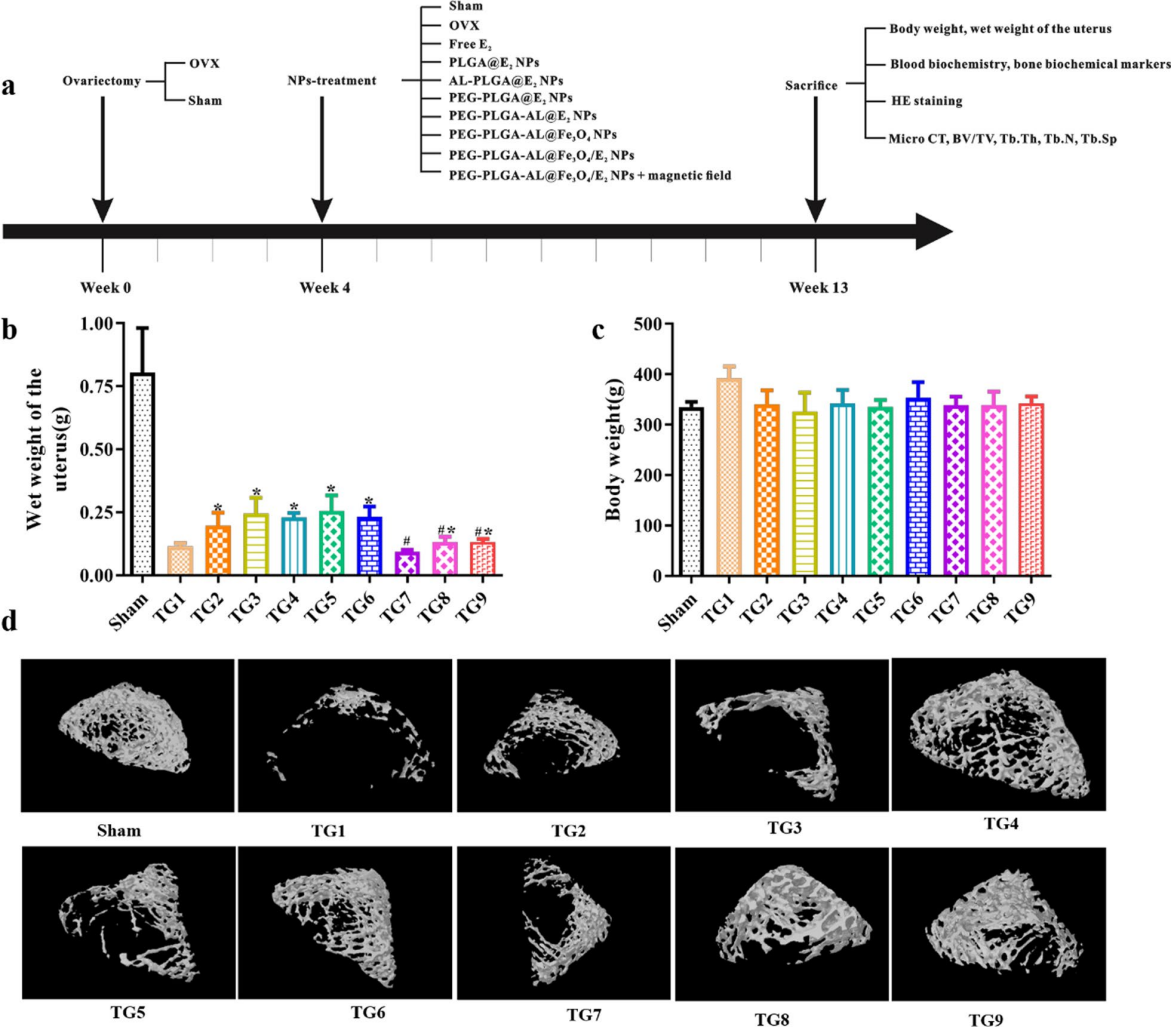
Treatment effect of NPs. **a** The treatment protocol. **b** Plots of body weight at week 9 after treatment (n = 8). **c** Plot of uterine wet weight in different groups at week 9 after treatment (n = 9). *P < 0.05 versus free E_2_, ^#^*P* < 0.05 versus PEG-PLGA-AL@E_2_ NPs. **d** Representative three-dimensional trabecular architecture of the distal femur from the respective groups obtained by micro-CT examination at week 9 after treatment. Treatment groups TG1: OVX, TG2: free E_2_, TG3: PLGA@E_2_ NPs, TG4: AL-PLGA@E_2_ NPs, TG5: PEG-PLGA@E_2_ NPs, TG6: PEG-PLGA-AL@E_2_ NPs, TG7: PEG-PLGA-AL@Fe_3_O_4_ NPs, TG8: PEG-PLGA-AL@Fe_3_O_4_/E_2_ NPs, TG9: PEG-PLGA-AL@Fe_3_O_4_/E_2_ NPs + MF

To evaluate the safety and therapeutic effect of NPs, we used OVX rats as an animal model. The treatment protocol was depicted in Fig. 8a. There was no statistic difference in body weight among all the groups (Fig. 8b). In order to analyze the effect of NPs on the uterus, the final uterine wet weights were recorded. As seen in Fig. 8c, the final wet weights of the uteruses in the OVX group were approximately 15% of those in the sham group. Meanwhile, uterine wet weight was statistically higher in the free E_2_-treated group than that of the OVX group. Uterine wet weights of animals in the PEG-PLGA-AL@Fe_3_O_4_ NPs, PEG-PLGA-AL@Fe_3_O_4_/E_2_ NPs and PEG-PLGA-AL@Fe_3_O_4_/E_2_ NPs + MF groups were significantly lower than in the PEG-PLGA-AL@E_2_-treated group.

For sake of evaluating the bio-safety of NPs, we analyzed the serum markers and the histopathology of liver, tibia, uterus and small intestine. Results of hematological study (Additional file 1: Figure S3) showed that WBC, RBC, HGB and PLT counts in the rats showed no significant differences from those of control. There were also no significant changes in serum ALT, AST, ALP, Cr and TC among all groups (Additional file 1: Figure S4). As can be seen from the H&E results (Additional file 1: Figure S5), there were no significant pathological changes between the sham, OVX and the other treatment groups. Hence, we concluded that 3 months exposure to any of the NPs did not contribute to apparent toxic effects on OVX rats.

### Effects of the NPs on bone mineral density and bone microarchitecture

**Fig. 9:**
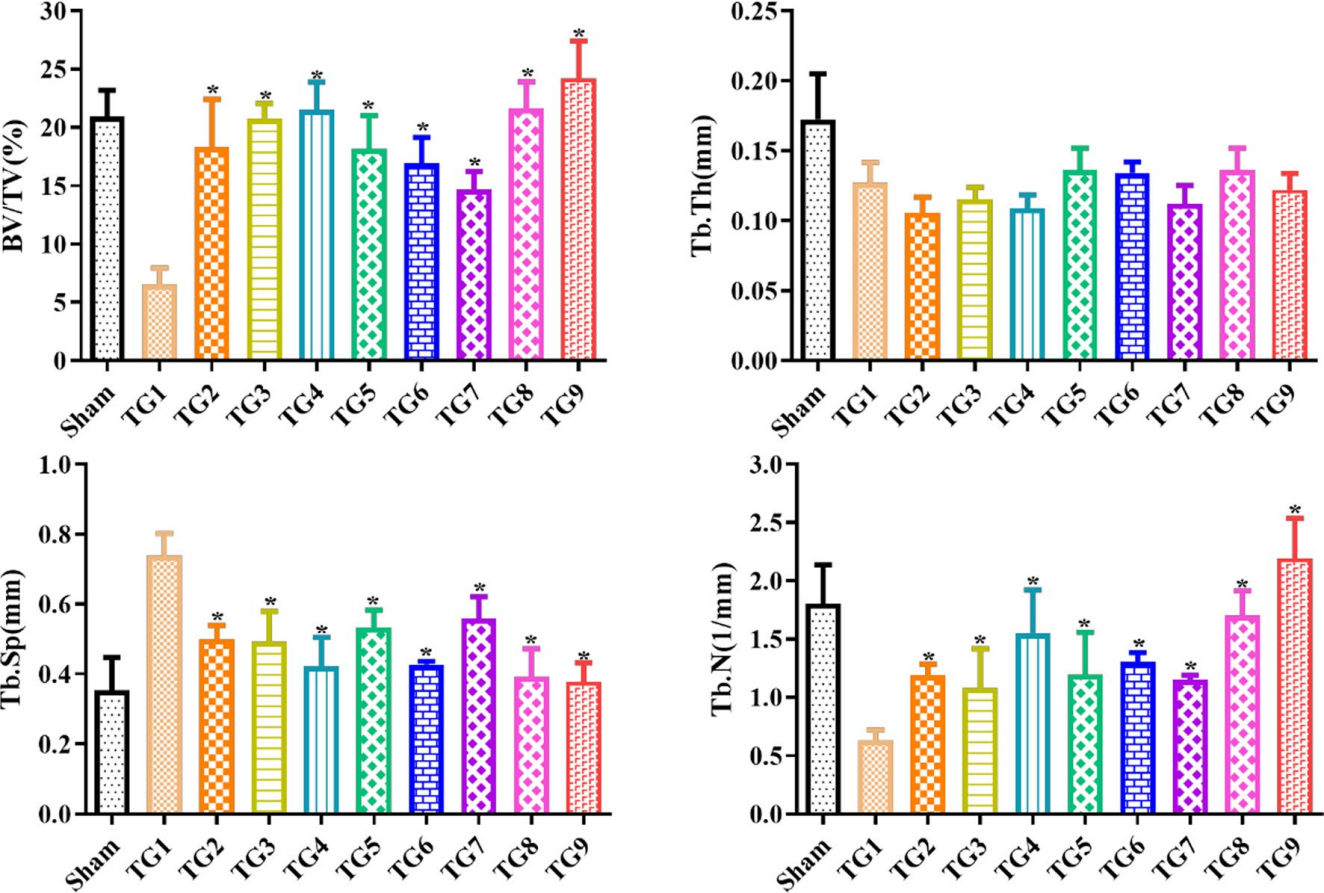
The percent bone volume (BV/TV), trabecular thickness (Tb.Th), trabecular separation (Tb.Sp) and trabecular number (Tb.N) as measured by micro-CT in OVX rats (n = 9). *P < 0.05 versus OVX. Treatment groups TG1: OVX, TG2: free E_2_, TG3: PLGA@E_2_ NPs, TG4: AL-PLGA@ E_2_ NPs, TG5: PEG-PLGA@E_2_ NPs, TG6: PEG-PLGA-AL@E_2_ NPs, TG7: PEG-PLGA-AL@Fe_3_O_4_ NPs, TG8: PEG-PLGA-AL@Fe_3_O_4_/E_2_ NPs, TG9: PEG-PLGA-AL@Fe_3_O_4_/E_2_ NPs + MF

In order to analyze the extent of bone loss induced by NPs, the trabecular bone volume of distal femurs and proximal tibias was evaluated by micro-CT (Fig. 8d). The sham group showed a 3.21-fold higher percentage BV/TV than the OVX group (*P* < 0.01, Fig. 9), while the PEG-PLGA-AL@Fe_3_O_4_/E_2_ + MF NPs treatment group exhibited a 2.72-fold increase in BV/TV compared with the OVX group (*P* < 0.01). The increase in BV/TV of the PEG-PLGA-AL@Fe_3_O_4_/E_2_ NPs-treated group was merely 145% compared to the PEG-PLGA-AL@E_2_ NPs treated group (P < 0.05). Furthermore, we measured the microarchitecture parameters. In accordance with the results of BV/TV, the Tb.N in the OVX group showed significantly lower than that of sham group. Otherwise, the Tb.Sp in the OVX group was higher than that of the sham. A changeover was seen in the group of PEG-PLGA-AL@Fe_3_O_4_/E_2_ + MF, reducing Tb.Sp by 51.35% compared to the OVX group (*P* < 0.05). The numerical value of Tb.Sp was approximate to the value showed in sham group. Thus, these results implied that treatment with PEG-PLGA-AL@Fe_3_O_4_/E_2_ + MF effectively improved OVX-induced OP in rats.

### NPs improve femoral bone strength in OVX rats

**Fig. 10:**
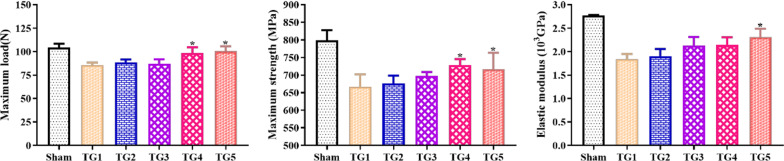
Effects of the flavonoids of NPs on biomechanical properties of the left femur in OVX rats (n = 8). ^*^P < 0.05 *vs*. the OVX group. Treatment groups TG1: OVX, TG2: PEG-PLGA-AL@E_2_ NPs, TG3: PEG-PLGA-AL@Fe_3_O_4_ NPs, TG4: PEG-PLGA-AL@Fe_3_O_4_/E_2_ NPs, TG5: PEG-PLGA-AL@Fe_3_O_4_/E_2_ NPs + MF

Furthermore, we studied the bone strength of the right femur by adopting the three-point bending test. The right femurs showed marked decreases in the mechanical strength index consisting of maximum load, maximum strength and elastic modulus in the OVX group (*P* < 0.05, Fig. 10). Meanwhile, this decrease in the mechanical strength index was reversed by treatment with NPs. Particularly, the mechanical strength indexes were statistically higher in the PEG-PLGA-AL@Fe_3_O_4_/E_2_ NPs + MF group compared to the OVX group. The results of bone strength about other different groups did not reach to any statistically significant degree.

### NPs affected expression of bone turnover markers

**Fig. 11:**
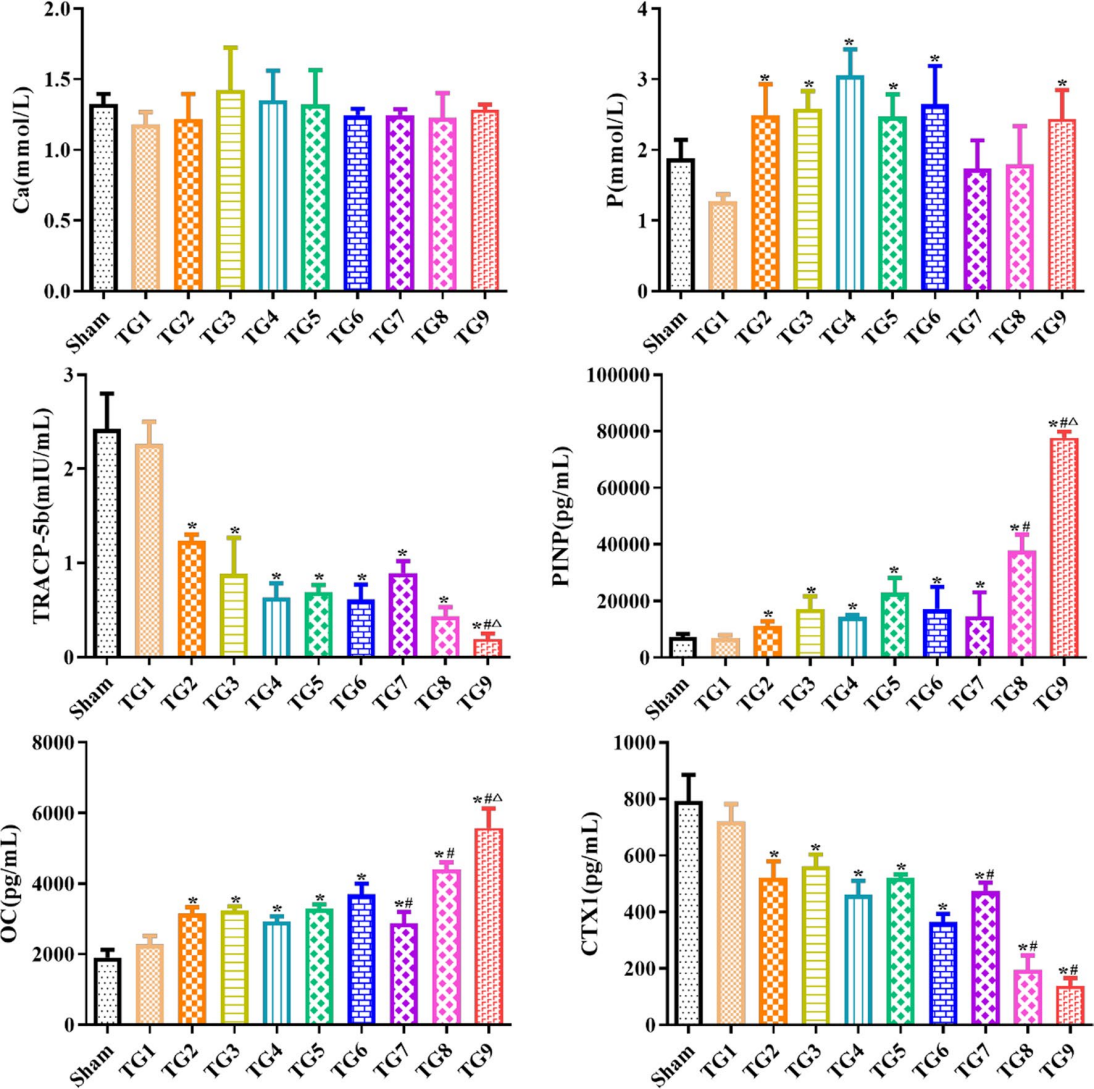
The effect of NPs on serum Ca, P, TRACP-5b, PINP, OC, and CTX1 at end of study (week 13, n = 9). ^*^P < 0.05 versus Sham; ^#^*P* < 0.05 versus PEG-PLGA-AL@E_2_ NPs; ^△^*P* < 0.05 versus PEG-PLGA-AL@Fe_3_O_4_/E_2_ NPs. Treatment groups TG1: OVX, TG2: free E_2_, TG3: PLGA@E_2_ NPs, TG4: AL-PLGA@E_2_ NPs, TG5: PEG-PLGA@E_2_ NPs, TG6: PEG-PLGA-AL@E_2_ NPs, TG7: PEG-PLGA-AL@Fe_3_O_4_ NPs, TG8: PEG-PLGA-AL@Fe_3_O_4_/E_2_ NPs, TG9: PEG-PLGA-AL@Fe_3_O_4_/E_2_ NPs + MF

In order to further clarify the anti-OP mechanism of NPs, we analyzed the expression of bone turnover markers representing bone formation and resorption (Fig. 11). The results showed that drug-treated groups exhibited significantly upregulated serum expression of PINP and OC compared with the sham and OVX groups (*P* < 0.05). The values of CTX1 and TRAP-5b in the drug-treated groups were markedly decreased compared with the sham and OVX groups (*P* < 0.01). Moreover, compared with other drug-treated groups, NPs-treatment caused changed trends and a statistically-significant change in all these bone turnover markers, except CTX1, was observed in the PEG-PLGA-AL@Fe_3_O_4_/E_2_ NPs + MF group. Compared with the groups treated with NPs except PEG-PLGA-AL@Fe_3_O_4_/E_2_ NPs, CTX1 was noticeably decreased in the group treated with PEG-PLGA-AL@Fe_3_O_4_/E_2_ NPs + MF (*P* < 0.05). There were no significant effects on the concentrations of Ca or P in the blood.

### NPs induced new bone formation in the proximal femur

**Fig. 12:**
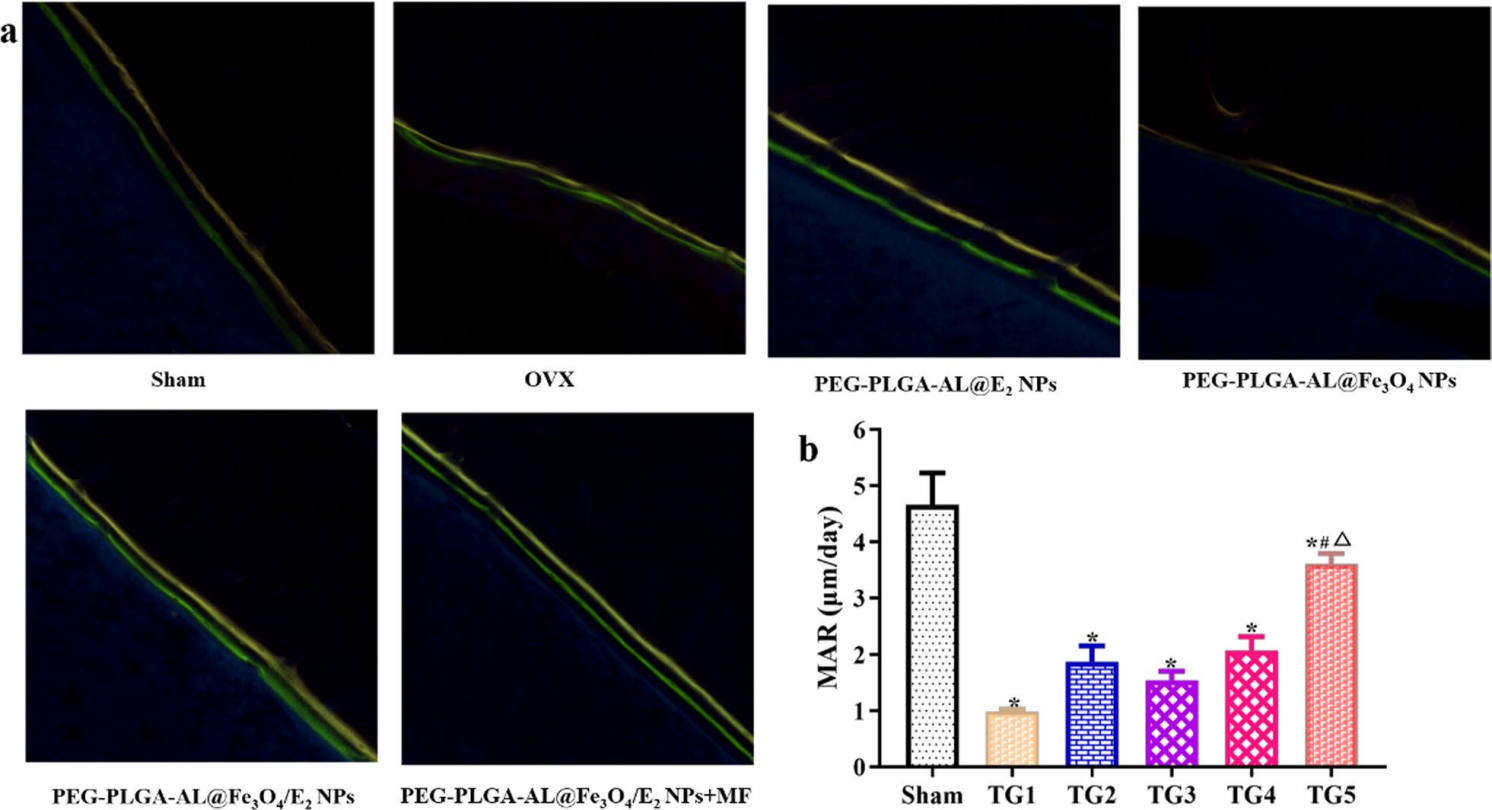
Effects of NPs on new bone formation. **a** Representative images of the right tibial regions from different groups. **b** The distance between the double fluorescence labeled lines (n = 3). Treatment groups TG1: OVX, TG2: PEG-PLGA-AL@Fe_3_O_4_ NPs, TG3: PEG-PLGA-AL@Fe_3_O_4_ NPs, TG4: PEG-PLGA-AL@Fe_3_O_4_/E_2_ NPs, TG5: PEG-PLGA-AL@Fe_3_O_4_/ E_2_ NPs + MF. ^*^P < 0.05 versus Sham; ^#^*P* < 0.05 versus PEG-PLGA-AL@E_2_ NPs; ^△^*P* < 0.05 versus PEG-PLGA-AL@Fe_3_O_4_/E_2_ NPs

To assess new bone formation, tetracycline/calcein double labeling was used to detect dynamic bone formation in vivo and measure histomorphometric parameters. Representative fluorescent images from the different drug formulation-treated groups are shown in Fig. 12. The images show that both calcein (green) and tetracycline (yellow) were detected in the same regions on the bone surface of the drug-treated rats. The distance between the tetracycline and calcein labels in the model group was noticeably shorter than that of the sham group. In contrast, the distance between the tetracycline and calcein labels of the NP-treated groups was increased. As these results showed, treatment with PEG-PLGA-AL@ Fe_3_O_4_/E_2_ enhanced new bone formation.

## Discussion

Bone is a dynamically-remodeled organ that is continuously maintained in a balance between bone resorption (the breakdown and removal of old bone tissue by osteoclasts) and bone formation (new bone formation by osteoblasts). OP is one of the bone diseases resulting from an imbalance between bone formation and bone resorption [30]. ERT plays an important role in preventing and treating OP [31]. E_2_, the most well-known steroid hormone, has been widely used as a drug of HRT to prevent and treat OP. However, because of the poor tissue selectivity and the side effects, the clinical application of E_2_ has been recognized as unsatisfactory in the last decade [32]. For sake of overcoming this problem, there has been a more urgent than ever need for researchers to develop new generations of drug delivery systems to prevent and treat OP. Thus, bone-targeting E_2_ therapy should be taken into consideration to facilitate OP treatment. In this study, we constructed a remote-controlled release system based on bone-targeted multi-functional NPs, to deliver E_2_ to treat PMOP.

PLGA, one of the FDA-approved copolymers, is composed of lactic acid and glycolic acid and is well known as a drug delivery system [33]. Due to the excellent biocompatibility, processability, adjustable biodegradability, controlled degradation time, suitable mechanical strength and cargo loading adaptability, PLGA and PLGA-based materials have been widely used in artificial bone-substitute [34], such as PLGA-based engineered three-dimensional scaffolds [35, 36], PLGA/HA/PLA-AP/phBMP-4 composite scaffold [37] and polymer fiber scaffolds [38]. We adopted the most frequently-used PLGA copolymer with a lactide/glycolide molar ratio of 50:50 for the preparation of NPs. To prolong circulation, PEGylation of nanocarriers was used to reduce opsonization and subsequent phagocytosis by the reticuloendothelial system (RES). In order to enhance the drug concentration in the targeted sites, most researchers modify the surface of the drug delivery system with specific molecules [29]. The molecules most frequently employed for targeting bone tissue are bisphosphonates [39], tetracycline [40], acidic oligopeptides [41] and E_2_ analogs [42]. In the present study, AL, a representative bisphosphonate, was a suitable bone-targeting moiety for its present clinical use. AL was conjugated to the carboxyl group of acid-terminated PLGA polymer to target the bioactive agent to the bone tissue. Fe_3_O_4_ NPs have attracted enormous attention and are used in various biomedical applications on account of their advantages, such as biological nontoxicity, chemical stability and cost-effectiveness [43]. However, the drawbacks limiting their application are their spontaneous aggregation and vessel embolism after intravenous application. A strategy to address this problem is the combination of Fe_3_O_4_ with polymers to improve the stability and biocompatibility of Fe_3_O_4_ NPs [44]. According to our previous research, a magnetothermally-triggered drug delivery system has been verified in polymer capsules loaded with Fe_3_O_4_, which could facilitate immediate, massive drug release [24]. Therefore, magnetic fluid hyperthermia was exploited to induce drug release of estrogen from PLGA NPs encapsulating 10 nM Fe_3_O_4_ in this work.

The NPs formulations were synthesized through the emulsion solvent diffusion evaporation method with a hydrodynamic diameter of around 200 nm and a negative surface charge, which was in line with previous reports [24]. The NPs showed good stability, good biocompatibility and high encapsulation ability for E_2_. The magnetic properties confirmed that the magnetism of Fe_3_O_4_ NPs and the magnetism of the material hardly varied. Further external heating experiments showed that the release of NPs is temperature dependent. These results confirmed that temperature played a critical role in drug release, and the amount of drug released increased at higher temperatures, which was consistent with other carrier systems reported before [24]. Employing an appropriate vector is an important issue in developing a bone-targeting delivery system [45]. Based on the above information, the co-loaded NPs could be an extremely promising method of bone-targeted delivery. Cellular uptake in monocytes indicated that Fe_3_O_4_-loaded NPs were effectively taken up by murine pre-osteoclastic Raw 264.7 cells. The biodistribution testified that the co-loaded NPs had a good effect on the enrichment of drugs in bone tissue compared with other NPs. The results also revealed that the AL-conjunction had a certain degree of bone-targeting ability.

Nowadays, the OVX-induced OP model is the most widely-adopted animal model used to simulate the pathogenesis of PMOP. The major characteristics of this model are decreased bone quality, uterine atrophy, reduced uterine weight and enhanced bone resorption [46]. In accordance with the previous study, uterine weight was reduced in OVX-rats compared with the sham [47]. Next, we validated the in vivo safety of the drug delivery system. The serum biomarker assay revealed no differences between treatment groups or between NP-treated and control groups. No remarkable changes were observed between NP-treated and sham groups in the liver, uterus or small intestine. The results of histological analysis of these organs were in accordance with serum biomarker results. These data suggested that the NPs exerted no apparent toxic effects in this study according to hematological index and histological analysis.

One of the characteristics of OP is the loss of trabecular bone and deterioration of bone architecture. Hence, we used 3D micro-CT imaging to evaluate the potential effects of NP treatment on bone microstructure. The OVX group showed declines of BV/TV, Tb.Th and Tb.N, accompanied by increased Tb.Sp. These experimental phenomena implied the occurrence of bone loss and deterioration. However, the trend was reversed in NP-treated animals, with values consistent with those of sham group. These results demonstrate that co-loaded NPs can improve OVX-induced OP through increased BV/TV, and decreased Tb.N and Tb.Sp. In addition, the three-point bending test is usually adopted to detect bone strength by measuring mechanical loads on bones. As expected, the results showed that NP treatment improved bone strength.

Bone turnover markers change more sensitively than bone mineral density, so a number of researchers analyzed bone turnover markers to analyze therapeutic effects of OP treatments. These markers reflect bone formation and bone resorption [48]. Indeed, an increased risk of fracture is generally accompanied by an increase in bone turnover markers [49]. Therefore, we analyzed levels of the bone turnover markers PINP, OC, CTX1 and TRAP-5b in serum. PINP is an extracellular catabolite from bone collagen type I [50]. OC is a specific non-collagenous bone matrix protein secreted by mature osteoblasts, while PINP and OC are non-specific collagen proteins, mainly produced by osteoblasts. Therefore, the determination of OC and PINP content in blood can reflect the activity of osteoblasts. CTX1 is a fragment of collagen type I decomposed during bone maturation, which can sensitively reflect the activity of osteoclasts and is considered to be a specific indicator of bone resorption [50]. TRAP-5b is one of the acid phosphatase isoenzymes that are specifically expressed in osteoclasts and is a specific and highly-sensitive indicator of bone resorption [49]. As expected, PINP and OC in the NP-treated groups were significantly increased, whereas CTX1 and TRAP-5b were downregulated in the NP-treated groups. As a consequence, we quantitatively assessed bone formation using double labelling with tetracycline and calcein. Quantitative measurements showed that new bone formation increased, which was consistent with the results of analysis of bone turnover markers.

AL, the most commonly-used third-generation bisphosphonate, binds to hydroxyapatite (HA) in the bone, inhibiting osteoclastic bone resorption and thus suppressing bone loss. Various studies have clarified the role of AL in enhanced bone mineral density and weakened bone turnover markers [47, 51]. Although estrogen has an important impact in maintaining bone mass, significant side effects of estrogen, such as the increasing risk of oncogenicity and cardiovascular diseases, cannot be ignored [52]. This drug delivery system co-loaded E_2_ and Fe_3_O_4_ to prevent OP in OVX rats and aimed to a magnetically remote-controllable drug release through external heat. Our data showed that the NPs inhibited bone resorption and enhanced bone formation, with the benefit of no obvious toxic side effects. These data suggest that combined application of E_2_ and bisphosphonates should be considered and that this therapy would be beneficial for reducing the dose and frequency in treatment of OP.

## Conclusion

In conclusion, this study successfully constructed a remote-controlled release system based on bone-targeted multi-functional NPs by the emulsion solvent diffusion method to deliver E_2_ and Fe_3_O_4_ to treat OP. The NPs showed strong stability, good biocompatibility and high encapsulation ability. The remote-controllable release of E_2_ from PLGA-AL NPs demonstrated an excellent magnetism-responsive property. The NPs had a certain degree of bone-targeting ability and circulation time was increased after modification with AL and PEG. Importantly, this multi-functional delivery system ameliorated OVX-induced bone loss, improved bone strength and induced new bone formation with less side effects on non-skeletal tissues. Our study provided evidence to support the use of a remote-controlled release system based on bone-targeted multifunctional NPs for anti-OP. These findings could provide a new strategy for bone-targeted therapy of OP.

## Supplementary Information

**Additional file 1**: **Table S1**. Encapsulation efficiency (EE%) and drug loading efficiency (DLE%) of estradiol in PLGA@E_2_ NPs, AL-PLGA@E_2_ NPs, PEG-PLGA@E_2_ NPs and PEG-PLGA-AL@E_2_ NPs. **Table S2**. EE% and DLE% of estradiol in PEG-PLGA-AL@E_2_ NPs. **Table S3**. The characteristics of PEG-PLGA-AL@ Fe_3_O_4_/E_2_ NPs. **Figure S1**. Synthesis of AL-PLGA. Synthetic scheme (a) and associated ^1^H-NMR spectra of PLGA (b) and AL-PLGA (c). **Figure S2**. In vitro particle stability assay of PEG-PLGA-AL@Fe_3_O_4_/E_2_ NPs in 1×PBS or FBS. **Figure S3**. The effect of NPs on WBC, RBC, HGB and PLT at end of study (week 13). **Figure S4**. The effect of NPs on ALT, AST, ALP, Cr and TC in sham and OVX rats at end of study (week 13). **Figure S5**. Sections of main organs were obtained and stained with H&E.

## Data Availability

All data used and analyzed during this study are available from this published article and its supplementary information files. The datasets used and analyzed during the current study are available from the corresponding author on reasonable request.
