# Supplementary material for: Remote-controllable bone-targeted delivery of estradiol for the treatment of ovariectomy-induced osteoporosis in rats
Source: J Nanobiotechnology. 2021 Aug 18;19:248. doi: 10.1186/s12951-021-00976-4 (PMC8371851; doi:10.1186/s12951-021-00976-4)
Supplement: Supplementary file 1 — Additional file 1: Table S1. Encapsulation efficiency (EE%) and drug loading efficiency (DLE%) of estradiol in PLGA@E2 NPs, AL-PLGA@E2 NPs, PEG-PLGA@E2 NPs and PEG-PLGA-AL@E2 NPs. Table S2. EE% and DLE% of estradiol in PEG-PLGA-AL@E2 NPs. Table S3. The characteristics of PEG-PLGA-AL@ Fe3O4/E2 NPs. Figure S1. Synthesis of AL-PLGA. Synthetic scheme (a) and associated 1H-NMR spectra of PLGA (b) and AL-PLGA (c). Figure S2. In vitro particle stability assay of PEG-PLGA-AL@Fe3O4/E2 NPs in 1×PBS or FBS. Figure S3. The effect of NPs on WBC, RBC, HGB and PLT at end of study (week 13). Figure S4. The effect of NPs on ALT, AST, ALP, Cr and TC in sham and OVX rats at end of study (week 13). Figure S5. Sections of main organs were obtained and stained with H&E. [file 12951_2021_976_MOESM1_ESM.docx]

**Supplementary Information**

# Remote-controllable bone-targeted delivery of estradiol for the treatment of ovariectomy-induced osteoporosis in rats

Yuanyuan Guo, Yongwei Liu, Chen Shi, Tingting Wu, Yongzhi Cui, Siyuan Wang, Ping Liu, Xiaobo Feng, Yu He& Dehao Fu

**Table S1**. Encapsulation efficiency (EE%) and drug loading efficiency (DLE%) of estradiol in PLGA@E_2_ NPs, AL-PLGA@E_2_ NPs, PEG-PLGA@E_2_ NPs and PEG-PLGA-AL@E_2_ NPs; data represent the mean and standard deviation (n = 3).

| Groups | PLGA@E_2_ NPs | AL-PLGA@E_2_ NPs | PEG-PLGA@E_2_ NPs | PEG-PLGA-AL@E_2_ NPs |
| --- | --- | --- | --- | --- |
| EE% | 72.01 ± 2.61 | 62.64 ± 7.13 | 64.74 ± 16.55 | 58.31 ± 9.17 |
| DLE% | 1.43 ± 0.05 | 1.24 ± 0.14 | 1.24 ± 0.31 | 1.15 ± 0.18 |

**Table S2**. EE% and DLE% of estradiol in PEG-PLGA-AL@E_2_ NPs.

| Ratio of PLGA and E_2_ | 25:0.5 | 25:1 | 25:1.5 |
| --- | --- | --- | --- |
| EE% | 58.34 ± 9.21 | 40.41 ± 6.82 | 46.41 ± 1.39 |
| DLE% | 1.15 ± 0.18 | 1.72 ± 0.28 | 2.66 ± 0.08 |

**Table S3**. The characteristics of PEG-PLGA-AL@ Fe_3_O_4_/E_2_ NPs.

| Ratio of PLGA and Fe_3_O_4_ | 25:0.25 | 25:0.5 | 25:0.75 |
| --- | --- | --- | --- |
| Diameter/nm | 180.01 ± 3.1 | 181.42 ± 1.2 | 182.03 ± 2.0 |
| PDI | 0.25 ± 0.04 | 0.24 ± 0.01 | 0.25 ± 0.02 |
| Zeta potential/mv | -4.68 ± 0.20 | -4.18 ± 0.80 | -4.66 ± 0.30 |
| E_2_ EE% | 67.39 ± 4.41 | 65.78 ± 8.42 | 51.44 ± 9.41 |
| E_2_ DLE% | 1.30 ± 0.08 | 1.27 ± 0.16 | 1.00 ± 0.18 |
| Fe_3_O_4_ EE% | 56.10 ± 6.11 | 57.81 ± 4.63 | 57.42 ± 4.64 |
| Fe_3_O_4_ DLE% | 0.55 ± 0.06 | 1.12 ± 0.09 | 1.66 ± 0.13 |


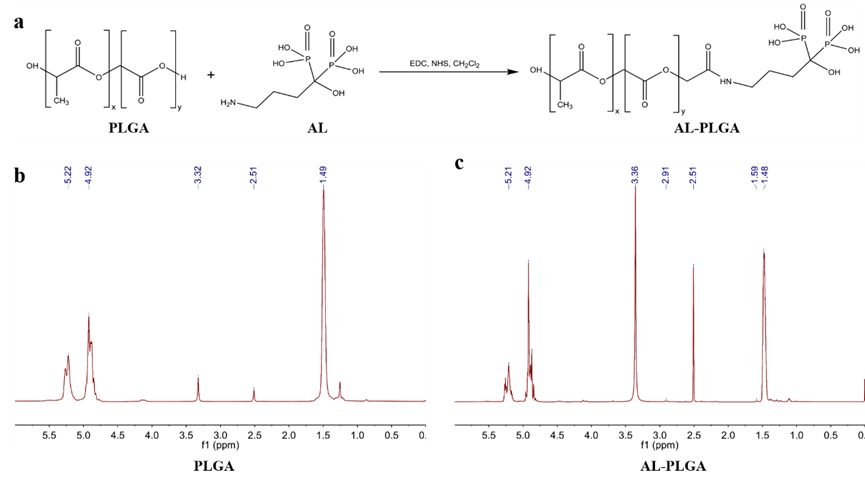


**Figure S1**. Synthesis of AL-PLGA. Synthetic scheme (**a**) and associated ^1^H-NMR spectra of PLGA (**b**) and AL-PLGA (**c**) (600 MHz, DMSO-d6).


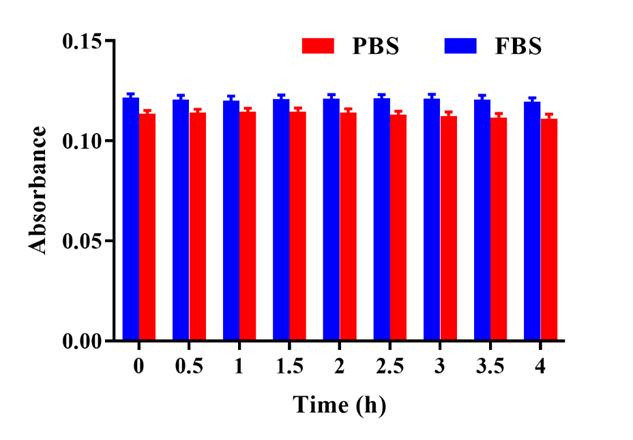


**Figure S2**. In vitro particle stability assay of PEG-PLGA-AL@Fe_3_O_4_/E_2_ NPs in 1×PBS or FBS (n = 3).


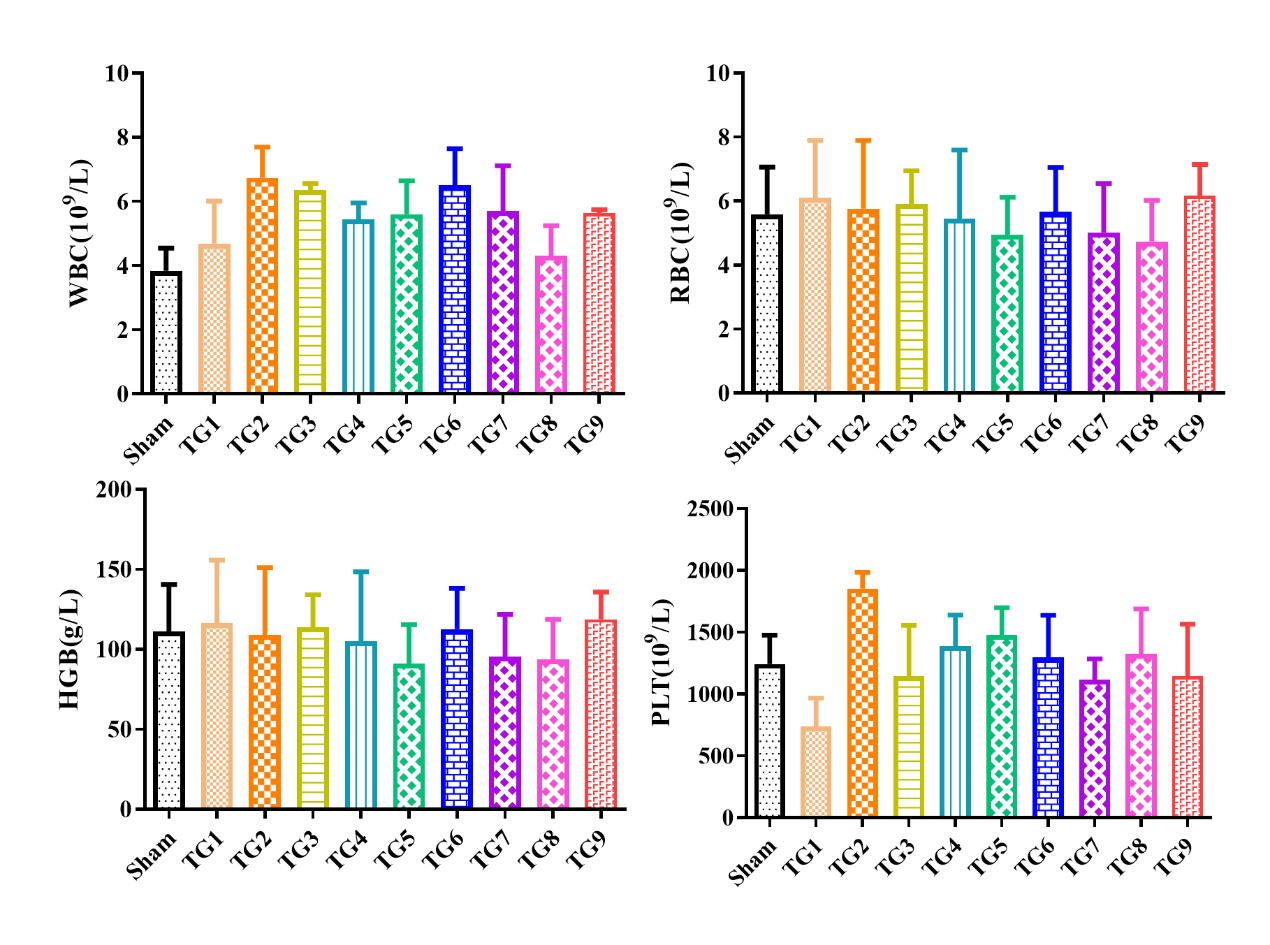


**Figure S3**. The effect of NPs on WBC, RBC, HGB and PLT at end of study (week 13). No significant differences were found between treatment groups (*P* > 0.05, n = 8). Treatment groups TG1: OVX, TG2: free E_2_, TG3: PLGA@E_2_ NPs, TG4: AL-PLGA@E_2_ NPs, TG5: PEG-PLGA@E_2_ NPs, TG6: PEG-PLGA-AL@E_2_ NPs, TG7: PEG-PLGA-AL@Fe_3_O_4_ NPs, TG8: PEG-PLGA-AL@Fe_3_O_4_/E_2_ NPs, TG9: PEG-PLGA-AL@Fe_3_O_4_/E_2_ NPs +MF.


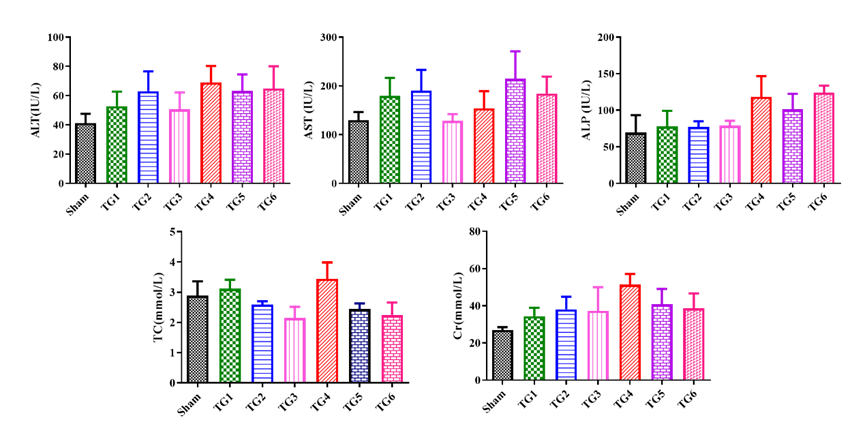


**Figure S4**. The effect of NPs on ALT, AST, ALP, Cr and TC in sham and OVX rats at end of study (week 13). No significant differences were found between treatment groups (*P* > 0.05, n = 8). ALT: alanine aminotransferase, AST: aspartate aminotransferase, ALP: alkaline phosphatase, Cr: creatinine, TC: total cholesterol. Treatment groups TG1: OVX, TG2: free E_2_, TG3: PLGA@ E_2_ NPs, TG4: PEG-PLGA@E_2_ NPs, TG5: PEG-PLGA-AL@E_2_ NPs, TG6: PEG-PLGA-AL@Fe_3_O_4_/E_2_ NPs.


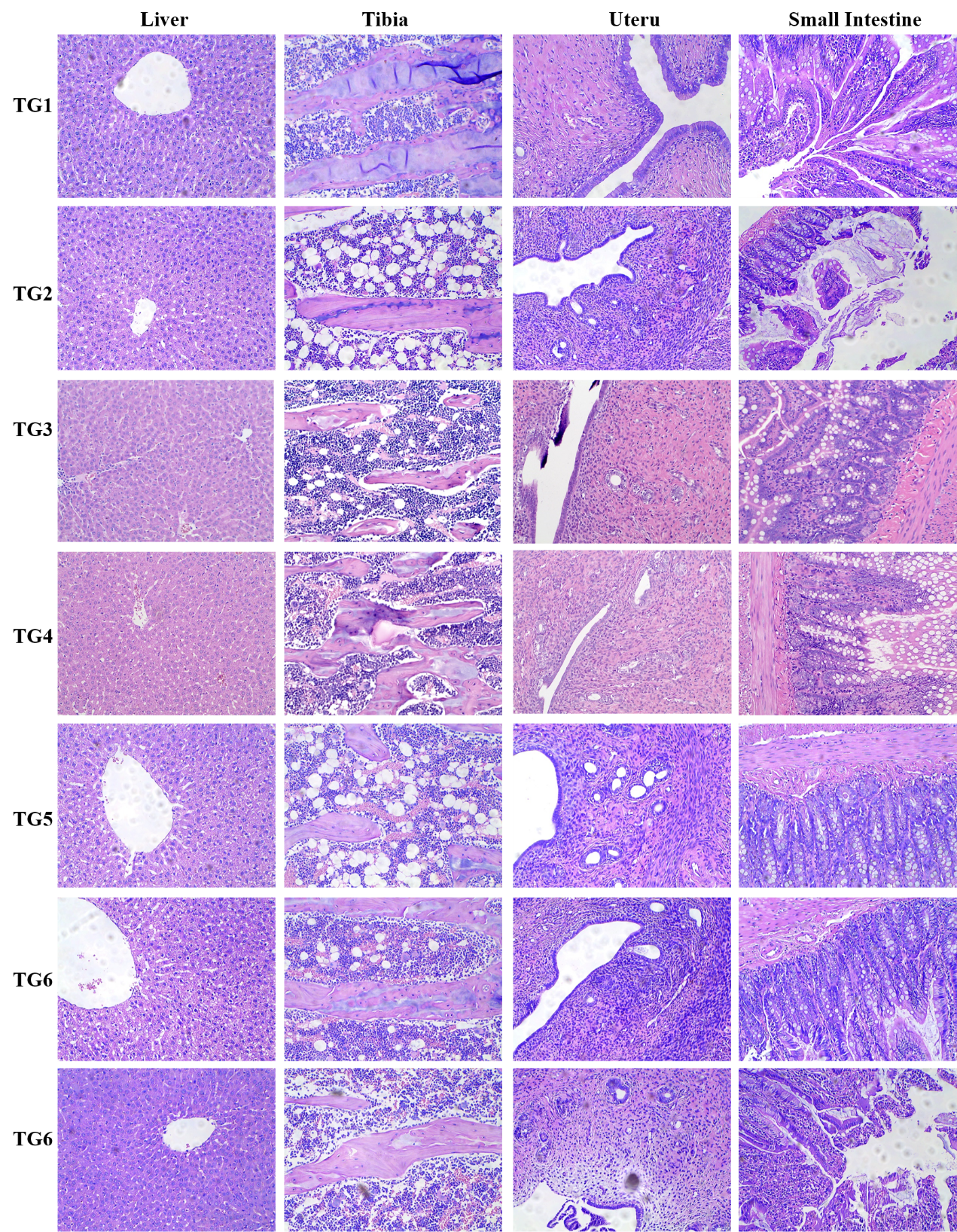


**Figure S5**. Sections of main organs were obtained and stained with H&E. Treatment groups TG1: OVX, TG2: free E_2_, TG3: PLGA@ E_2_ NPs, TG4: PEG-PLGA @ E_2_ NPs, TG5: PEG-PLGA-AL@E_2_ NPs, TG6: PEG-PLGA-AL@Fe_3_O_4_/ E_2_ NPs.
